# A plant tethering system for the functional study of protein-RNA interactions in vivo

**DOI:** 10.1186/s13007-022-00907-w

**Published:** 2022-06-04

**Authors:** Diego Cuerda-Gil, Yu-Hung Hung, Kaushik Panda, R. Keith Slotkin

**Affiliations:** 1 Donald Danforth Plant Science Center, St. Louis, MO USA; 2 Department of Molecular Genetics, The Ohio State University, Columbus, OH USA; 3 Division of Biological Sciences, University of Missouri, Columbia, MO USA

## Abstract

The sorting of RNA transcripts dictates their ultimate post-transcriptional fates, such as translation, decay or degradation by RNA interference (RNAi). This sorting of RNAs into distinct fates is mediated by their interaction with RNA-binding proteins. While hundreds of RNA binding proteins have been identified, which act to sort RNAs into different pathways is largely unknown. Particularly in plants, this is due to the lack of reliable protein-RNA artificial tethering tools necessary to determine the mechanism of protein action on an RNA in vivo. Here we generated a protein-RNA tethering system which functions on an endogenous Arabidopsis RNA that is tracked by the quantitative flowering time phenotype. Unlike other protein-RNA tethering systems that have been attempted in plants, our system circumvents the inadvertent triggering of RNAi. We successfully in vivo tethered a protein epitope, deadenylase protein and translation factor to the target RNA, which function to tag, decay and boost protein production, respectively. We demonstrated that our tethering system (1) is sufficient to engineer the downstream fate of an RNA, (2) enables the determination of any protein’s function upon recruitment to an RNA, and (3) can be used to discover new interactions with RNA-binding proteins.

**Supplementary Information:**

The online version contains supplementary material available at 10.1186/s13007-022-00907-w.

## Introduction

Plant genomes encode hundreds of proteins that interact with and regulate RNA [20]. However, the roles of these proteins in post-transcriptional gene regulation remain widely unknown, in part due to the lack of experimental tools to study their function. For example, it is not understood which proteins are sufficient for the key regulatory decision that directs an RNA transcript to enter either the RNA decay or RNA interference (RNAi) pathway [14]. This decision point is critical, as decay will only remove one RNA transcript, while the positive feedback cycle of RNAi carries the fate of continued degradation of additional RNA molecules through the production of small interfering RNAs (siRNAs) [33]. Artificially recruiting a protein of interest to a known RNA in vivo (protein-RNA tethering) is an essential technique for deciphering the function of RNA-binding proteins. Once artificially forced to a reporter RNA, the unknown function of the protein on that RNA can be assessed by standard RNA and protein biology techniques. Systems such as bacteriophage *MS2-MCP* (*MS2* coat protein binds an RNA sequence called the *MS2* stem-loop) and boxB-λN (λN protein binds an RNA sequence called box B) have been used in yeast, *Drosophila* and other systems to tether a protein to a reporter RNA in order to study mRNA stability, splicing, localization, transport and translation [5]. More recently a CRISPR/Cas system has been discovered that uses a CRISPR guide RNA (gRNA) to program the targeting of the Cas13 protein to an RNA, rather than the typical DNA target of Cas9 [2]. Protein-RNA tethering can be accomplished by synthetically fusing a nuclease-dead version of Cas13 to any protein-of-interest to investigate the function of that protein-of-interest on the RNA (reviewed in [35]).

Plants are highly sensitive to the production of double-stranded RNA (dsRNA) (reviewed in [14]). Whether it is via transcription through an inverted repeat (forming an intramolecular hairpin), the pairing of complementary transcripts (intermolecular interaction) or produced by an RNA-dependent RNA Polymerase (RDR) protein, dsRNA is a trigger for RNA cleavage by DICER family proteins [31, 32]. This cleavage produces either a single small RNA molecule (microRNA) or if the dsRNA is longer, a series of siRNAs, both of which are able to trigger post-transcriptional gene silencing (PTGS) of complementary mRNA transcripts (reviewed in [33]). In some cases, the cleaved target mRNA is further converted into dsRNA by an RDR protein and produces secondary siRNAs in the cycle of RNAi, amplifying the PTGS and resulting in significant reduction of complementary mRNAs and their encoded proteins [10, 34].

Existing protein-RNA tethering systems are not well-developed in plants because they each trigger the plant's sensitive dsRNA response. In the case of the *MS2-MCP* and boxB-λN systems, they both require the target RNA to be transgenic in order to carry the necessary *MS2* stem-loop or box B binding sites. The hairpin dsRNA secondary structure of these binding sites closely resembles stem-loop structures normally processed by DICER family proteins [5, 24]. In plants, use of these *MS2* stem-loop and box B binding sites complicates downstream analyses, as transgenic reporter RNAs are often subject to PTGS even without protein tethering [13, 21]. Cas13 systems of protein-RNA tethering can overcome this problem, as they can target any endogenous RNA and are not dependent on the formation of intramolecular dsRNA [25]. However, CRISPR gRNAs need to be complementary to their target RNA and subsequent base pairing will generate 28–30 nucleotide (nt) intermolecular dsRNA [2]. This gRNA base pairing to the target RNA is known in plants to trigger PTGS of the target RNA even without the presence of the Cas13 protein [28]. Therefore, each of the existing in vivo systems of protein-RNA tethering trigger the plant’s sensitive response to dsRNA, degrading the target RNA independently of protein binding or action. In order to identify new RNA-binding proteins and characterize their function, we aimed to generate a novel plant in vivo protein-RNA tethering system in which the target RNA is stable and not subject to PTGS. Here we describe a protein-RNA tethering system that acts on an endogenous (non-transgenic) RNA without intramolecular or intermolecular dsRNA formation, and consequently does not spontaneously trigger PTGS.

## Results

### A minimal version of the BRN1 protein retains *SOC1* RNA-binding

**Fig. 1:**
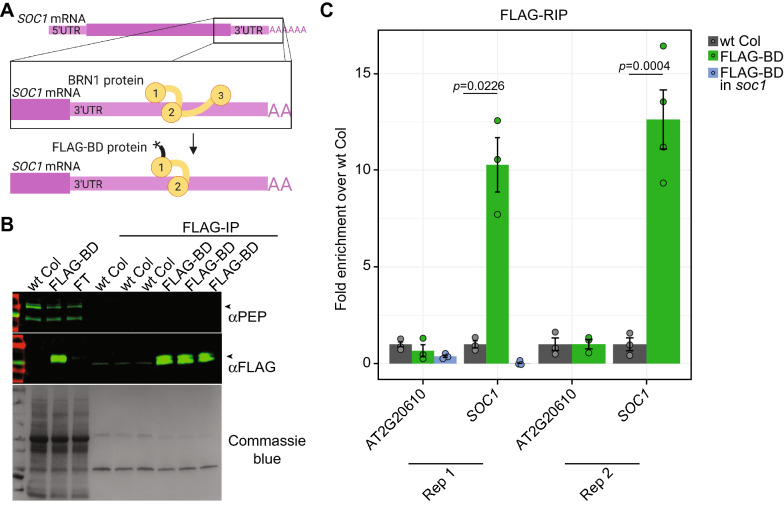
The epitope-tagged minimal RNA-binding protein ‘FLAG-BD’ binds the *SOC1* mRNA. **A** The Arabidopsis BRN1 protein contains three RRM domains (1–3) that bind the *SOC1* mRNA 3’UTR [18]. Only two RRM domains (1–2) are necessary for *Bruno*-like proteins to bind their targets [29]. The BRN1 protein inhibits *SOC1* translation [18], and this is thought to be mediated via the protein region between RRM 2 and 3. We generated a FLAG epitope-tagged (asterisk) truncated BRN1 protein with only RRM domains 1 and 2 (FLAG-BD, bottom). Figure created with BioRender. **B** Western blot of the FLAG-immunoprecipitation in plants with and without the FLAG-BD transgene. The three wt Col and FLAG-BD samples are biological replicates. PEP is an unrelated protein used as a loading control. FT = Flow Through fraction unbound to the FLAG antibody. Arrowheads mark the predicted size of the protein detected. **C** FLAG-IP followed by RNA extraction and qRT-PCR of samples from (**B**). AT2G20610 is an unrelated gene used as a negative control. Each biological replicate is shown as a circle. The bar represents the average and error bars represent the standard deviation between three or more biological replicates. P-value is calculated by using an unpaired t-test with Welch's correction. The RIP experiment was repeated twice (Rep 1 / Rep 2) using distinct biological replicate plants

*Bruno*-like proteins are deeply conserved RNA-binding proteins. In Drosophila, *Bruno* binds a repeated 7-nt sequence in the 3' UTR of the *Oskar* mRNA [29]. In *Arabidopsis thaliana*, the *Bruno* ortholog *Bruno*-like 1 (BRN1) binds a single 7-nt sequence (5’UAUGUAU) in the 3'UTR of the *SOC1* mRNA (Fig. 1A) and limits *SOC1* translation [18]. SOC1 is a known integrator of flowering time cues, as *soc1* mutant plants flower late and *brn1* mutants have the opposite effect of higher accumulation of SOC1 protein and flower early [15, 18]. Although *Bruno*-like proteins characteristically have three RNA Recognition Motif (RRM) domains (domains 1–3, Fig. 1A), across several species only the first two RRM binding domains (BDs) are necessary for mRNA interaction and specificity [1, 8, 12, 29]. We generated a FLAG-tagged minimal Arabidopsis BRN1 protein that contains only the first two RRM domains (named ‘FLAG-BD’, Fig. 1A), excluding the unnecessary third RRM and the region that putatively functions to inhibit *SOC1* translation. We generated stable transgenic plants with an integrated FLAG-BD transgene driven by the viral 35S promoter in the wild-type Columbia (wt Col) background and confirmed its protein production (Fig. 1B). We next immunoprecipitated the FLAG-BD protein in three biological replicates (Fig. 1B) and performed an RNA-immunoprecipitation (RIP) experiment followed by qRT-PCR. In two distinct experiments with different biological replicates, we found a 10.6-fold and 12.5–fold enrichment of the *SOC1* mRNA compared to wt Col plants without FLAG-BD (Fig. 1C). As an additional control, we transformed the same FLAG-BD transgene into the *soc1* mutant background and did not detect *SOC1* mRNA in our RIP of FLAG-BD (Fig. 1C). This experiment confirms that the minimal FLAG-BD protein retains the ability to bind the endogenous *SOC1* mRNA.

In addition to *SOC1*, there are 2236 other mRNAs in the Arabidopsis transcriptome that have the identical 7-nt BRN1 binding site in their 3’UTR. To test if FLAG-BD also binds these RNAs, we focused on 3 mRNAs that are similarly expressed as the *SOC1* mRNA in the leaf tissue under examination. Although the variation is high, we found that FLAG-BD binds these other mRNAs in addition to the *SOC1* mRNA (Additional file 1: Figure S1). The promiscuity of FLAG-BD binding may reflect the broad binding of the endogenous BRN1 protein to many or all mRNAs with the 7-nt binding site, which has not been investigated on a transcriptome-wide level.

### Protein tethering itself does not trigger PTGS or alter regulation of the *SOC1* RNA

**Fig. 2:**
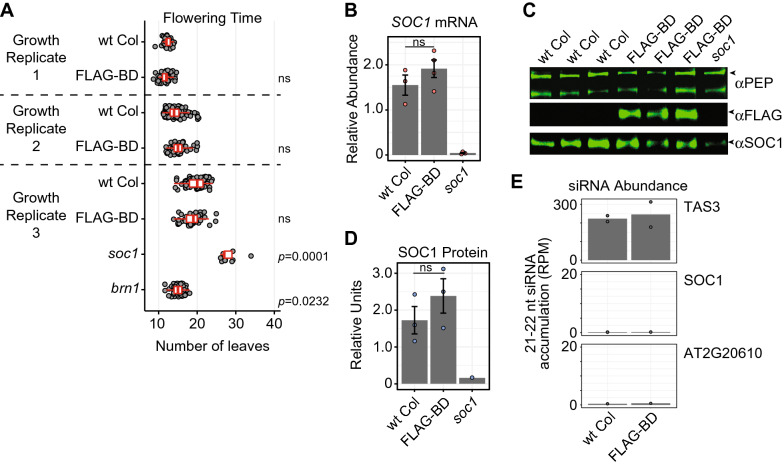
FLAG-BD binding does not alter SOC1 regulation. **A** Flowering time is measured as the number of leaves generated at the time the first flower opens. Gray points are individual plants, and the red box plots represent the 25th and 75th percentiles of the sample population, with the center bar representing the median and whiskers at the 10th and 90th percentile. P-values are comparisons to the wt Col in the same growth replicate, calculated by using unpaired t-test. ns = not statistically significant. **B** qRT-PCR of *SOC1* mRNA levels in plants with and without FLAG-BD. Three or more biological replicates for each genotype were used (shown as red points), the height of the bar represents their average and the error bars represent the standard deviation. P-value is calculated by using unpaired t-test with Welch’s correction. **C** Western blot displaying SOC1 protein levels between biological replicates with and without FLAG-BD. **D** SOC1 protein quantification from the Western blot in part (**C**). Biological replicate data points are shown as blue points, the height of the bar represents their average and error bars represent the standard deviation. P-value is calculated by using unpaired t-test with Welch’s correction. **E** Accumulation of siRNAs from wt Col and FLAG-BD lines. *TAS3* is a *trans*-acting siRNA producing locus shown as a positive control for siRNA accumulation. AT2G20610 is an unrelated gene without small RNA production used as a negative control. Two biological replicates are shown as points, and their average is the height of the bar. RPM = reads per million sequenced small RNAs

Using multiple lines of evidence, we found that the binding of the FLAG-BD protein does not impact SOC1 regulation. In three growth replicates, FLAG-BD plants flower at the same time as plants without the FLAG-BD transgene, while *soc1* and *brn1* mutants flower late and early, respectively (Fig. 2A) [15, 18]. If FLAG-BD triggered PTGS of *SOC1*, we would expect a reduction in *SOC1* mRNA and protein levels. Instead, we found that *SOC1* mRNA (Fig. 2B) and protein levels (Fig. 2C, D) are not decreased in plants with FLAG-BD. We did observe a small increase in the level of *SOC1* mRNA and protein in FLAG-BD plants, but this increase was not statistically significant (Fig. 2B and D). Importantly, FLAG-BD tethering does not trigger siRNA production from the *SOC1* mRNA when assayed by small RNA sequencing (Fig. 2E), again demonstrating that the *SOC1* mRNA is not entering PTGS. Therefore, FLAG-BD is a novel protein tool that can be used as a protein-RNA tethering system to the endogenous *SOC1* mRNA, eliminating the issues from techniques previously developed outside of and moved into plants.

### Using FLAG-BD to discover new interacting proteins

**Fig. 3:**
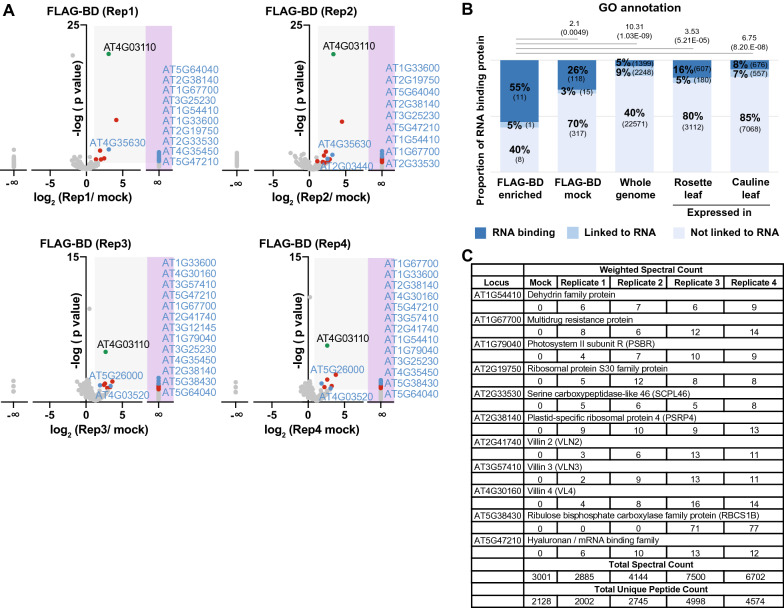
RNA-binding proteins interact with FLAG-BD. **A** Volcano plots of anti-FLAG immunoprecipitation followed by Mass Spectrometry (IP-MS) of four biological replicates (Rep1-4). The x-axis shows the log2 fold change between each FLAG-IP sample and the mock-IP control, and the y-axis depicts the p-value of Fisher exact test in a negative log scale. The gray shaded region represents the region of statistical significance (fold change ≥ 2, p ≤ 0.05), while the pink shaded region represents proteins that accumulated in the FLAG-IP but did not accumulate in the mock-IP, making their enrichment value infinite. The green point indicates the bait protein BRN1. Red points indicate the significantly enriched proteins. Blue points indicate the proteins significantly enriched in at least 2 of the 4 biological replicates. Gels of the IP protein sample before Mass Spectrometry are shown as Additional file 1: Figure S2. **B** Stacked bar graph showing the RNA-interaction annotation of the 20 proteins enriched in at least 2 of the 4 biological replicates from part (**A **). These are compared to the annotation of the proteins identified in the mock-IP (FLAG-BD mock), the annotation of the entire Arabidopsis genome, or the genes annotated as expressed in rosette or cauline leaves. Bold indicates the percent of genes, with the total number of genes in parentheses. The hypergeometric test results are shown above each bar as fold enrichments (top number) and p-value in parentheses. **C** Table showing the 11 proteins previously designated as ‘RNA-binding’ enriched in at least 2 of the 4 FLAG-BD IPs from part (**B **). The weighted spectral count (number of spectra associated with only a specific protein group plus the apportioned number of spectra shared with other proteins) is indicated for each protein. The total spectral count and total unique peptide count is listed below. Additional results from the IP-MS experiment are shown in Additional file 2: Table S1

There are multiple methods to identify new proteins that interact during RNA binding, and some of these methods are specific to mRNAs or even RNAs with specific sequences [3, 5, 20]. To take advantage of the interaction between the epitope-tagged FLAG-BD and its target RNAs, we aimed to determine if FLAG-BD could be used to identify new interacting proteins. We performed four biological replicate immunoprecipitations (IPs) of FLAG-BD plants with anti-FLAG bound beads or a mock negative control with beads but no linked antibody (control gels shown in Additional file 1: Figure S2), and subjected these samples to liquid chromatography -coupled Mass Spectrometry (LC-MS). As expected, we found abundant spectra for the portions of the BRN1 protein that compose FLAG–BD (green points, Fig. 3A). We identified a number of other significantly enriched proteins in our data (blue and red points, Fig. 3A), and limited our analysis to 20 proteins that are significantly enriched in at least 2 of the 4 biological replicates (blue points, Fig. 3A). The identity and spectral counts of these 20 proteins is provided in Additional file 2: Table S1. 11 of these 20 proteins have been previously designated as ‘RNA-binding’ proteins or ‘linked to RNA’ [3], which represents a significant enrichment compared to the mock immunoprecipitation sample, the entire genome, or the predicted proteome of the leaf tissue that was examined (Fig. 3B). As a note, without a control that includes RNase, we cannot determine in our experiment if these proteins specifically bind RNA, as these proteins may interact directly with the FLAG-BD protein. The identity of the 11 proteins previously designated as ‘RNA-binding’, and their weighted spectral count in each biological replicate, are listed in Fig. 3C. This data demonstrates that the FLAG-BD tethering system can be used to identify new proteins that were previously unknown to interact during BRN1-RNA binding.

### Synthetic tethering of protein enzymatic activity to the *SOC1* mRNA

**Fig. 4:**
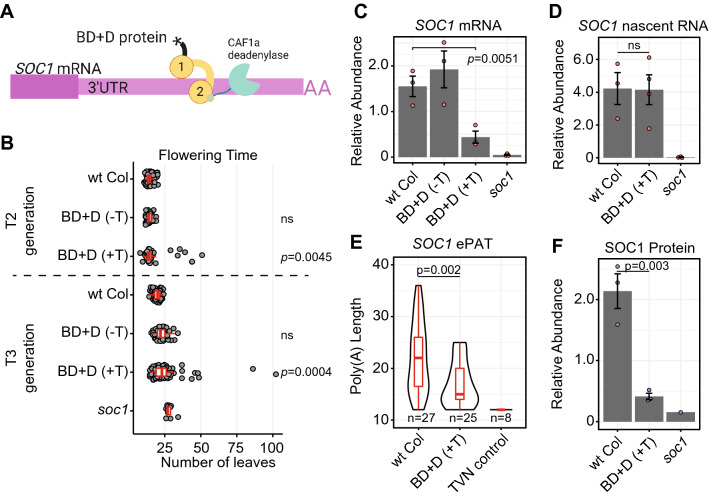
Proof-of-principle tethering of an RNA decay enzyme function to the *SOC1* mRNA. **A** Translational fusion between FLAG-BD and the CAF1a deadenylase protein generates the BD + D protein. **B **Flowering time of BD + D plants two (T2) and three (T3) generations after transformation. + T plants inherited the BD + D transgene, -T plants are siblings that did not inherit the transgene. Box plots and statistics are the same as in Fig. 2A. **C** qRT-PCR of *SOC1* polyadenylated mRNA. Three biological replicates of each genotype are shown as red points. Bar height, error bars and statistics are the same as Fig. 2B. **D** qRT-PCR of *SOC1* nascent transcripts (unspliced and not polyadenylated). Three or more biological replicates of each genotype are shown as red points. Bar height, error bars and statistics are the same as Fig. 2B. **E** ePAT assay to determine the poly(A) tail length of the *SOC1* mRNA. n = the number of clones Sanger sequenced. TVN is a control where the reverse transcription primer is anchored at the most 3’ nucleotide before the poly(A) tail begins. Box plot organization is the same as Fig. 2A. P-value is calculated by using an unpaired t-test with Welch’s correction. **F** Quantification of SOC1 protein accumulation in the BD + D line. Individual biological replicates are down as blue points. Bar height, error bars and statistics are the same as Fig. 2B

We next aimed to determine if proteins (and their enzymatic functions) could be artificially tethered to the *SOC1* mRNA using FLAG-BD. As a proof-of-principle, we generated a translational fusion of the Arabidopsis CAF1a deadenylase protein to the C-terminal end of FLAG-BD, generating the ‘BD + D’ protein (Fig. 4A). The CAF1a protein removes consecutive adenosine ribonucleotides from mRNA poly(A) tails [19], leading to RNA decay. Consequently, we predicted a destabilization of the *SOC1* mRNA and reduced protein production upon BD + D recruitment. We observed the late flowering phenotype that corresponds to the predicted decreased levels of SOC1 for 12% of second (T2) and third (T3) generation plants, while sibling plants that did not inherit the BD + D transgene do not display this phenotype (Fig. 4B). The incomplete penetrance of the late flowering phenotype in BD + D plants may be due to the reduced expression of the 35S: BD + D transgene compared to 35S: FLAG-BD (Additional file 1: Figure S3).

We next aimed to determine if the BD + D fusion protein was acting directly on the *SOC1* mRNA. In late flowering T2 BD + D plants, we detected the predicted decrease in *SOC1* mRNA levels (Fig. 4C). This reduction is purely post-transcriptional, as the level of nascent unspliced *SOC1* RNA is not altered (Fig. 4D). For these same late flowering BD + D plants, *SOC1* poly(A) tail length was directly assayed by ePAT [16] and Sanger sequencing of polyA tail products. We found that late flowering BD + D plants have a shorter distribution of *SOC1* mRNA poly(A) tail lengths compared to *SOC1* mRNAs in wt Col (Fig. 4E). As expected, the reduced level of *SOC1* mRNA and shorter poly(A) tail length corresponds to a decrease in SOC1 protein level (Fig. 4F, Additional file 1: Figure S4). Together, these data demonstrate that by using the BRN1 BD, a protein can be synthetically tethered to the endogenous *SOC1* mRNA, subject this mRNA to the protein’s enzymatic activity, and is sufficient to enhance the sorting of this RNA into the RNA decay pathway.

### Artificial protein tethering to an RNA can be used to increase protein production

**Fig. 5:**
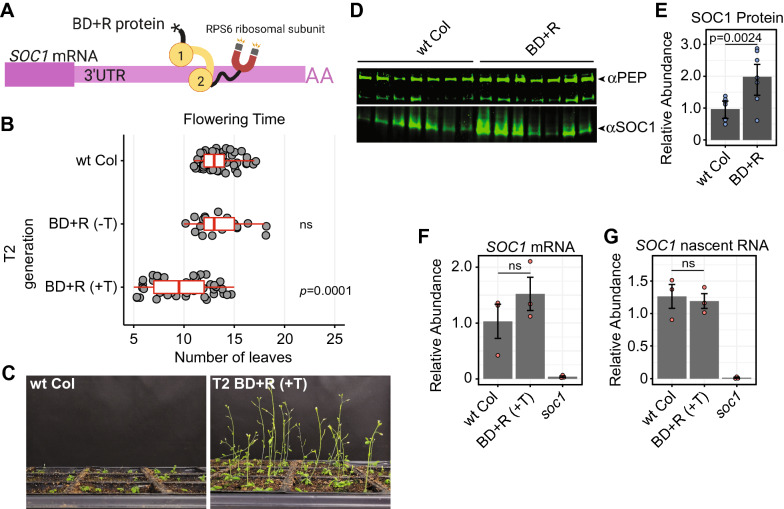
Tethering of a ribosomal protein translation factor to the *SOC1* mRNA boosts protein production. **A** Translational fusion between FLAG-BD and the RPS6 ribosomal protein generates the BD + R protein. **B** Flowering time of T2 BD + R plants grown side-by-side with wt Col. + T plants inherited the BD + R transgene, -T plants are siblings that did not inherit the transgene. **C** Representative images of T2 BD + R (+ T) plants grown side-by-side with wt Col taken 24 days after germination. **D** Western blot of SOC1 protein accumulation in seven biological replicates of early-flowering plants of the BD + R line compared to wt Col. PEP is an unrelated protein used as a loading control. **E** Quantification of SOC1 protein accumulation in the BD + R line from part (**D**). Individual biological replicates are shown as blue points. Bar height, error bars and statistics are the same as Fig. 2B. **F** qRT-PCR of *SOC1* polyadenylated mRNA. Three biological replicates of each genotype are shown as red points. Bar height, error bars and statistics are the same as Fig. 2B. **G** qRT-PCR of *SOC1* nascent transcripts (unspliced and not polyadenylated). Three biological replicates of each genotype are shown as red points. Bar height, error bars and statistics are the same as Fig. 2B

In Fig. 4 we targeted a reduction in *SOC1* mRNA levels, however, the programmed destruction of RNA (knockdown) can also be accomplished by transforming a plant with an artificial microRNA or siRNA-generating construct [4, 26]. In contrast to targeting RNA decay and degradation, increasing RNA translation and protein production is not easily programmed. Therefore, we aimed to tether a protein that would enhance mRNA translation and result in higher SOC1 protein level. Similar to the BD + D fusion protein, we generated a fusion between FLAG-BD and RPS6, a conserved protein of the 40S ribosomal subunit that enhances translation in *Arabidopsis* (generating ‘BD + R’) (Fig. 5A) [7]. In addition, because of the poor expression of the BD + D transgene and corresponding low penetrance of the BD + D late flowering phenotype (Fig. 4B, Additional file 1: Figure S3), we switched the promoter driving expression of BD from the viral 35S to the constitutive endogenous AtUBQ10 promoter for subsequent transgenes (see Methods). We found that the BD + R construct conferred the expected early flowering phenotype in 55% of T2 plants compared to either wt Col plants or siblings that did not inherit the transgene (Fig. 5B, C). These early-flowering plants display the expected higher accumulation of SOC1 protein (Fig. 5D, E). This increase in SOC1 protein in BD + R lines was determined to be the result of post-transcriptional and/or translational-level mechanisms, as the level of *SOC1* mRNA is only slightly higher (but not statistically significant, Fig. 5F) and unspliced nascent RNA is unaltered compared to wt Col (Fig. 5G). We conclude that protein fusions to the BRN1 BD result in the successful artificial tethering of enzymatic functions to the *SOC1* mRNA, and can be used to either increase or decrease SOC1 protein levels.

## Discussion

We have generated a synthetic protein-RNA tethering system that functions in vivo on an endogenous RNA, which can be monitored by the flowering time quantitative phenotype. We successfully and reproducibly tethered proteins to the *SOC1* mRNA, and in each instance demonstrated the utility of the fused protein. Importantly, this system was capable of sorting the *SOC1* mRNA into different fates, with CAF1a tethering leading to RNA decay and RPS6 tethering leading to enhanced translation. These proof-of-concept experiments prove that it is possible to synthetically tether a protein and enzymatic activity of the user’s interest to the *SOC1* mRNA. This system can be used to dissect the molecular function of RNA-interacting proteins, using *SOC1* as an endogenous reporter mRNA. To aid in the fusion of any protein of interest to FLAG-BD, we generated an AtUBQ10: FLAG-BD vector with a multiple cloning site to facilitate the insertion of the user’s protein of interest (Additional file 1: Figure S5D). We have made the sequences, plasmids and seed stocks of this protein-RNA tethering system available to the community (see Availability of data and materials).

Although the BRN1 BD—*SOC1* mRNA tethering system overcomes a key limitation of previous protein-RNA tethering systems in plants (triggering of PTGS), there are four limitations of this system. First, there is the complicating factor of the natural biology of the endogenous BRN1 protein. The endogenous BRN1 protein likely naturally binds more than one mRNA, and we find evidence of this promiscuity in our RIP data (Additional file 1: Figure S1). Therefore, in experiments such as our IP-MS (Fig. 3), we cannot be certain that the new proteins identified interact specifically with the *SOC1* mRNA. In the future, performing IP-MS with FLAG-BD in both wild-type and the *soc1* mutant plants would resolve this specificity issue. Second, the endogenous BRN1 protein likely competes with FLAG-BD for the 7-nt binding site in the *SOC1* 3'UTR. We see evidence of this competition in Fig. 2C, D when the levels of SOC1 protein are slightly elevated in FLAG-BD plants. In wt Col plants the normal binding of BRN1 to the *SOC1* 3'UTR results in translation repression [18], and this repression may be blocked by FLAG-BD occupying the binding site in *SOC1*. If this blocking occurs, it is not enough to generate a statistically significant change in protein levels (Fig. 2D) nor a phenotypic change in flowering time (Fig. 2A). If this blocking of the BRN1 binding site is a problem in the future, this could be overcome by performing FLAG-BD experiments without competition from the endogenous BRN1 protein in a *brn1* mutant background, although these plants would be expected to flower early (Fig. 2A). Third, we cannot be certain that the proteins annotated as ‘RNA-binding’ from our IP Mass Spectrometry experiment in Fig. 3 are actually binding an RNA. The ‘BD’ RNA-binding domain of the BRN1 protein may preclude the necessity or function of the RNA-binding domain located on the protein-of-interest when it is translationally fused with BD. We have not tested if adding BD interferes with RNA-binding domains of the fused protein-of-interest. In cases such as the identification of new proteins that interact with the BD-RNA binding module (Fig. 3), the specific RNA-binding of proteins should be individually validated by other methods. Fourth, in its current form, the FLAG-BD system cannot be used to investigate any RNA, but rather only the *SOC1* RNA and others that FLAG-BD binds. However, in the future this system could be used to change the fate of any exogenous transcript, including sorting into decay or increasing translation, by moving the *SOC1* 3’UTR or BRN1 binding site to a transgenic RNA.

## Methods

### Plant growth, propagation and collection

*Arabidopsis thaliana* plants of the *Columbia* (Col) ecotype were grown at 22°C on Pro-Mix FPX soil in Conviron MTPS-120 growth chambers in long days (16 h light / 8 h dark) with 200 µmol/m^2^/s light. Mutant alleles have been described previously and are shown in Additional file 3: Table S2. Transgenic lines were transformed by the Agrobacterium-mediated floral dip method and subsequently selected with Basta herbicide. For the production of T2 and T3 generations, T1 plants were pooled and self-fertilized without selection for flowering time phenotype. Leaf tissue was collected at the time of the opening of the first flower and was used for all experiments. Biological replicates are non-overlapping pools of individuals.

### Transgene production

The FLAG-BD and BD + D transgenes were synthesized by Integrated DNA Technologies (IDT) and cloned into pEarleyGate100 [9] using the restriction enzymes *Xho*I and *Xba*I. Maps of the plasmids and sequences are shown in Additional file 1: Figure S5.

To swap the 35S and AtUBQ10 promoters, the AtUBQ10 (AT4G05320) promoter + 5’UTR sequence from pICSL12015 [6] was directly amplified from wt Col genomic DNA using primers in Additional file 3: Table S2 that contain an additional sequence for In-Fusion Cloning (Takara). pDCG006 (Additional file 1: Figure S2) was digested with *BstB*I and *Xho*I to remove 35S, gel purified and In-Fusion recombined with the AtUBQ10 amplicon.

To facilitate protein fusions to FLAG-BD, we synthesized the "5’BD" cloning vector containing an ATG codon + 1X FLAG-epitope tagged minimal BRN1 protein + flexible linker sequence (no Stop codon) via IDT, leaving the MCS that originated in pEarleyGate100 intact for future cloning of proteins to be tethered. The resulting AtUBQ10:ATG-FLAG-BD-linker-MCS plasmid is called pDCG019 (map and sequence in Additional file 1: Figure S5D).

To generate the BD + R transgene, *RPS6* (AT4G31700) was amplified from wt Col genomic DNA using primers displayed in Additional file 3: Table S2, and In-Fusion cloned into pDCG019 digested with *Bam*HI and *Avr*II (map and sequence in Additional file 1: Figure S5C).

### Flowering time analysis

Flowering time was scored by counting the total number of rosette and cauline leaves of each plant at the time the first flower opened, as in [11]. Data for wt Col was collected repeatedly as it was grown side-by-side with the transgenic lines. Data was analyzed using *Rstudio* and plotted with *ggplot2*. P-value was calculated by using unpaired t-test.

### Western blotting

Leaf tissue was grounded in liquid nitrogen and thawed in lysis buffer (50 mM Tris–HCl pH 7.5, 150 mM NaCl, 5 mM MgCl_2_, 10% glycerol, 1% NP-40 (IGEPAL), 0.5 mM DTT, 1 mM PMSF, 1% Plant PIC (GoldBio protease inhibitor cocktail)) and homogenized for 15 min at 4°C. Lysates were clarified by centrifuging for 15 min at 4°C. Clarified lysates were reduced and denatured by boiling in 2X loading buffer at 95°C for 5 min, and then loaded onto 4%-20% gradient Tris–Glycine gels (BioRad). Proteins were separated at 200 V for 1 h. Protein was transferred from the gel to a PVDF membrane (Immobilon-FL, MilliporeSigma) using the BioRad semi-dry transblot for 35 min. Membranes were blocked for 1 h at room temperature in Odyssey/Intercept blocking buffer (LI-COR). Primary antibodies, which include anti-SOC1 (Agrisera), anti-PEP (Rockland), and anti-FLAG (Sigma Aldrich), were all diluted 1:2000 in Odyssey blocking buffer and incubated with blots overnight. The membranes were washed 5 times at room temperature with 1X PBS-T. The IR-800 Anti-rabbit secondary antibody (LI-COR) was diluted 1:5000 and incubated with membranes for 1 h. Membranes were washed 5 times at room temperature with 1X PBS-T, and then additional 2 times with 1X PBS. Blots were visualized using the Azure Sapphire Biomolecular Imager with exposure times ranging from 5 s to 5 min. Full images of un-cropped Western blots from all figures are shown in Additional file 1: Figure S6.

### Protein quantification

Digital images of Western blots were analyzed with *ImageJ* for relative pixel intensities. Non-specific background noise was subtracted from raw values. SOC1 protein quantification was calculated by the ratio of SOC1/PEP values. Biological replicates were averaged and the standard deviation was calculated using *Rstudio*. Significance was calculated with unpaired t-test with Welch’s correction.

### RNA immunoprecipitation

Before RNA-IP, 50 µl/IP of Dynabeads Protein G (Invitrogen) were washed in 1X PBS + 0.1% Tween, followed by incubation with 1 µg/IP FLAG antibody (Sigma) at room temperature for 90 min with rotation. For each sample, 0.5 g leaf tissue was crosslinked in formaldehyde and ground in liquid nitrogen. Proteins were extracted using 50 mM Tris–HCl pH 7.5, 150 mM NaCl, 5 mM MgCl_2_, 10% glycerol, 1% NP-40 (IGEPAL), 0.5 mM DTT, 1 mM PMSF, 1% Plant PIC (GoldBio protease inhibitor cocktail). Lysates were pre-cleared with Dynabeads Protein G (Invitrogen) with rotation for 20 min at room temperature. Pre-cleared lysates were then incubated with the prepared IP beads for 90 min at 4°C with rotation. Beads were washed 3X in the washing buffer (50 mM Tris–HCl pH 7.5, 150 mM NaCl, 5 mM MgCl_2_, 0.5 mM DTT). After the final wash, 1 mL Trizol LS (Invitrogen) per sample was added, reverse crosslinking was performed at 55°C for 5 min and RNA was extracted following the manufacturer’s protocol.

### Immunoprecipitation and mass spectrometry

Leaf tissue was crosslinked in 1% formaldehyde under vacuum for 3 min for a total of 5 times. Crosslinking was stopped by the addition of 200 mM glycine, then washed in water 5 times. Leaf tissue was ground to fine powder in liquid nitrogen using mortar and pestle. The powder was suspended in lysis buffer (20 mM Tris–HCl, pH 7.5, 5 mM MgCl2, 300 mM NaCl, 10% glycerol, 0.5 mM DTT, 1 mM PMSF, 0.1% IGEPAL, and 1% plant protease inhibitor (GoldBio)), then centrifuged for 10 min at 14,000 rpm at 4˚C. The supernatant was incubated with 50 µl of FLAG M2 magnetic beads (Sigma) at 4˚C for 3 h. The beads were then washed three times in cold TBS. The FLAG-IP was eluted twice with 50 µl of 0.1 M pH2.5 glycine and neutralized with 0.5 M Tris, 1.5 M NaCl pH8.0 solution.

FLAG-IP elutions were reduced (10 mM TCEP) and alkylated (25 mM Iodoacetamide) followed by digestion with Trypsin at 37°C overnight. The digest was acidified with 1%TFA before being cleaned-up with C18 tip. The extracted peptides were dried down and each sample was resuspended in 10 μL 5% ACN/0.1% FA. 5 μL was analyzed by LC–MS with a Dionex RSLCnano HPLC coupled to an Orbitrap Fusion Lumos (Thermo Scientific) mass spectrometer using a 2 h gradient. Peptides were resolved using 75 μm × 50 cm PepMap C18 column (Thermo Scientific).

All MS/MS samples were analyzed using *Mascot* (Matrix Science, London, UK; version 2.5.1.0). *Mascot* was set up to search against the provided sequences and the TAIR10 database. The digestion enzyme was set as trypsin. *Mascot* searched with a fragment ion mass tolerance of 0.60 Da and a parent ion tolerance of 10 ppm. Oxidation of methionine, carbamidomethylation of cysteine, and acetylation of N-terminal of protein were specified in *Mascot* as variable modifications.

*Scaffold* (4.8.2 Proteome Software Inc.) was used to validate MS/MS based peptide and protein identifications. Peptide identifications were accepted if they could be established under 1% FDR by the Peptide Prophet algorithm [17] with Scaffold delta-mass correction. Protein identifications were accepted if they could be established at greater than 99.0% probability and contained at least 2 identified peptides. Protein probabilities were assigned by the Protein Prophet algorithm [22]. Proteins that contained similar peptides and could not be differentiated based on MS/MS analysis alone were grouped to satisfy the principles of parsimony. Proteins sharing significant peptide evidence were grouped into clusters. Annotation of the proteins as RNA-binding, linked to RNA or not linked to RNA are from [3].

### RNA isolation

RNA was isolated using Trizol Reagent (Invitrogen) and RNA for RIP analysis was isolated using Trizol LS (Invitrogen) according to manufacturer instructions.

### Quantitative RT-PCR

5 µg of total RNA or the entire RIP RNA sample was DNase-treated using the Turbo DNA-free kit (Invitrogen). First-strand of cDNA synthesis, including the RIP RNA sample, was performed using an oligo-d(T) primer and Superscript IV reverse transcriptase (Invitrogen). For the detection of nascent RNAs, random hexamer primers were used for reverse transcription and one primer site is located in an intron. Primer sequences are shown in Additional file 3: Table S2. P-value was calculated using unpaired t-tests using Welch’s correction.

### Poly(A) tail length determination

The length of the poly(A) tail was determined by ePAT assay, performed as in [16]. Briefly, DNase-treated RNA was ligated to the ePAT anchor primer in Superscript III buffer supplemented with RNase Out (Invitrogen) and 5U Klenow Polymerase (New England Biolabs). 200U of Superscript III (Invitrogen) was added, and the solution was reverse transcribed at 55°C for 1 h. The cDNA was diluted 1:6 by adding 120 µl Elution Buffer. For the ePAT TVN control reaction, instead of ePAT anchor primer, the ePAT control primer was used. For PCR amplification of cDNA, primary PCR was performed with *SOC1* 3’UTR II Forward primer and ePAT anchor primer. For the ePAT TVN control sample, the ePAT control primer was used in place of the ePAT anchor primer. A nested PCR was performed by diluting the primary PCR 1:100 and repeating the PCR with the primer *SOC1* 3’UTR III Forward and the ePAT anchor primer. Amplicons were run on a 2% high resolution agarose gel and purified. Purified amplicons were TOPO TA cloned into pCR4-TOPO (Life Technologies) and transformed into *E. coli*. Plasmids from individual *E. coli* colonies were Sanger sequenced (Eton BioScience) and poly(A) tail length was analyzed in *Rstudio*. Primer sequences are shown in Additional file 3: Table S2. P-value was calculated using unpaired t-tests using Welch’s correction.

### Small RNA sequencing and analysis

100 µg of total RNA was enriched for small RNAs using the miRVana miRNA isolation kit (Life Technologies). 1 µg of enriched small RNA was used for library preparation with the TruSeq Small RNA Library Preparation Kit (Illumina). Multiplexed libraries were sequenced on an Illumina HiSeq 3000 at the Genome Technology Access Center in Washington University.

After sequencing, adapters were trimmed from raw sequences using fastx toolkit, t/rRNAs were removed and small RNAs were filtered to the 18–28 nt size range using *UEA small RNA Workbench* tool [30] and the small RNAs processed and normalized as described previously [23]. Small RNAs were mapped to the Arabidopsis TAIR10 genome using *Shortstack* [27] with default parameters except using the fractional-seeded guide approach for multi-mapped reads (–mmap f). *Rstudio* and *ggplot2* were used to generate siRNA graphs.

## Supplementary Information

**Additional file 1:** **Figure S1.** FLAG-BD interacts with other mRNAs in addition to *SOC1*. Extended analysis of the experiment from Figure 1C for three additional mRNAs that have the identical 7-nt BRN1 binding site sequence. Each biological replicate is shown as a point. The bar represents the average and error bars represent the standard deviation between three biological replicates. **Figure S2. **IP control gels performed before Mass Spectrometry. (A) Coomassie blue stained SDS-PAGE gels of the mock-IP (left) and four biological replicates (R1-R4) of the FLAG-IP (right). IN = input protein sample, FT = flow through sample that did not interact with the beads or FLAG antibody. (B) Western blot of the samples in part 'A'. The PEPC protein is not detected in the mock-IP or FLAG-IPs (top). The FLAG-BD protein is detected in the FLAG-IPs but not mock-IP (bottom). Arrowheads denote the expected size of the proteins detected. **Figure S3. **Reduced expression of the BD+D transgene. qRT-PCR of the relative mRNA accumulation of the FLAG-BD and BD+D transgenes. AT2G20610 is a constitutively-expressed control gene. At least three biological replicates for each genotype were used (shown as points), the height of the bar represents their average and the error bars represent the standard deviation. The transgene structure and position of the RT-PCR primers is shown above. **Figure S4. **SOC1 protein accumulation in BD+D plants. Western blot displaying SOC1 protein levels with the BD+D transgene. The three wt Col and BD+D samples are biological replicates. PEP is an unrelated protein used as a loading control. Arrowheads mark the predicted size of the protein detected. Quantification of this Western blot is shown in Figure 4F. **Figure S5.** Plasmid maps and sequences. Vector maps and annotated plasmid sequences of (A) FLAG-BD, (B) BD+D, (C) BD+R and (D) a vector with multiple cloning site (MCS) to fuse any protein to AtUBQ10:FLAG-BD. **Figure S6. **Full Western blots from other figures. The full un-cropped Western blot images from Figures 1B, 2C, 5D and S4.

**Additional file 2:** **Table S1. **Mass Spectrometry data of the 20 proteins identified in at least two biological replicates.

**Additional file 3:** **Table S2. **Primer sequences and alleles used in this study.

## Data Availability

Raw Illumina small RNA sequencing data from Fig. 2 produced for and analyzed in this study is available from NCBI GEO as GSE182403. The mass spectrometry proteomics data have been deposited to the ProteomeXchange Consortium via the PRIDE partner repository with the dataset identifier PXD031072 and 10.6019/PXD031072. Plasmid sequences are available in Additional file 1: Figure S5. Plasmids can be ordered without restriction from the corresponding author or Arabidopsis Biological Resource Center (stocks CD3-2286 to CD3-2289). Arabidopsis seeds of the lines described in this study are available without restriction from the corresponding author or Arabidopsis Biological Resource Center (stocks CS72796-CS72798).
