# Supplementary material for: A plant tethering system for the functional study of protein-RNA interactions in vivo
Source: Plant Methods. 2022 Jun 4;18:75. doi: 10.1186/s13007-022-00907-w (PMC9166424; doi:10.1186/s13007-022-00907-w)

Supplemental Figure 1

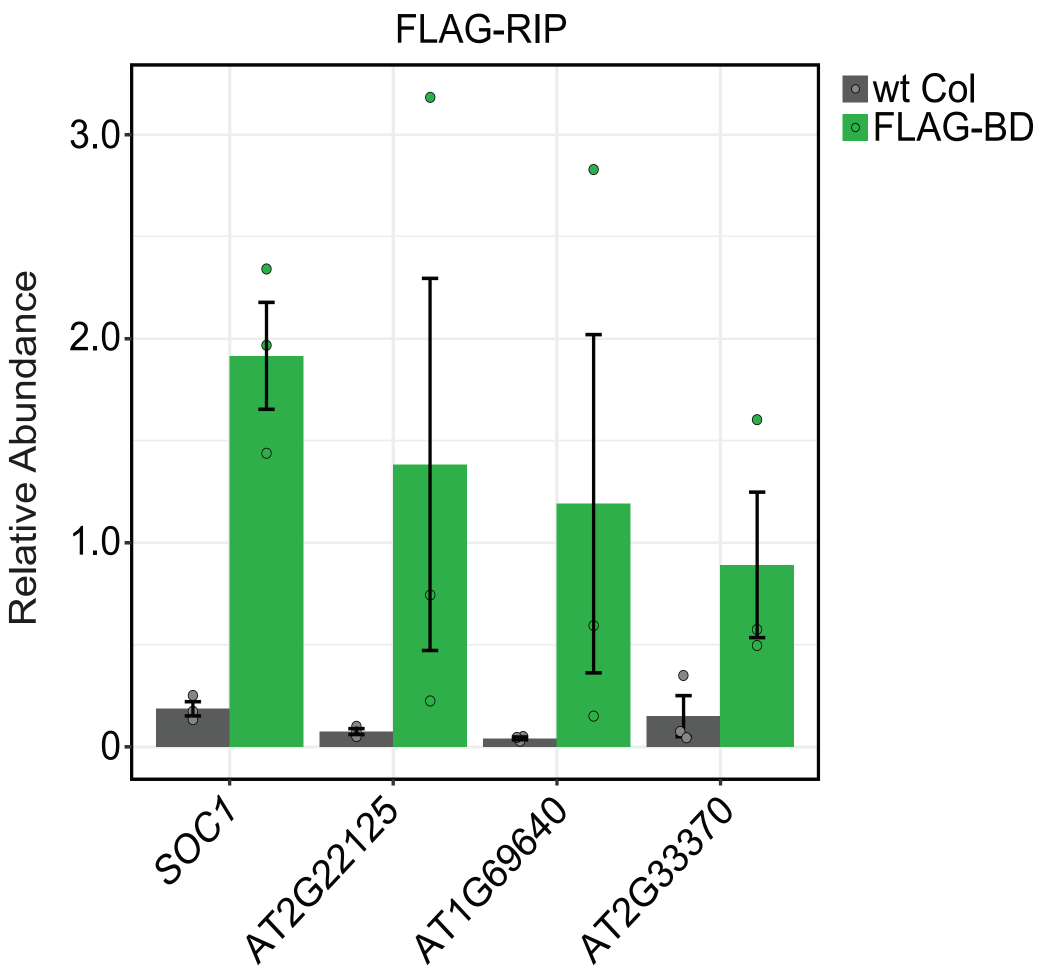

Supplemental Figure 2

A

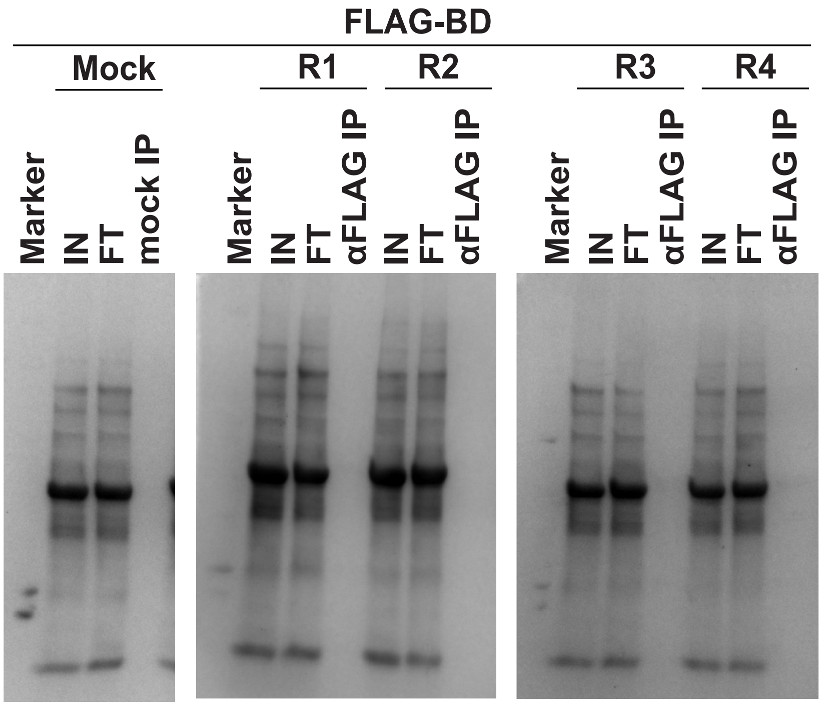

B

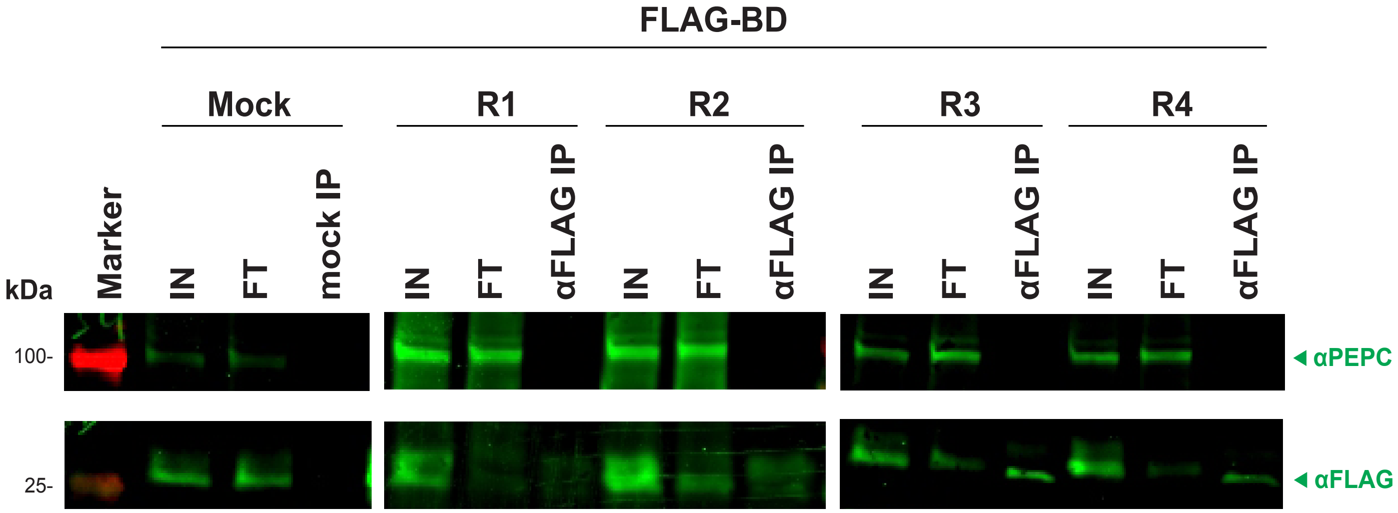

Supplemental Figure 3

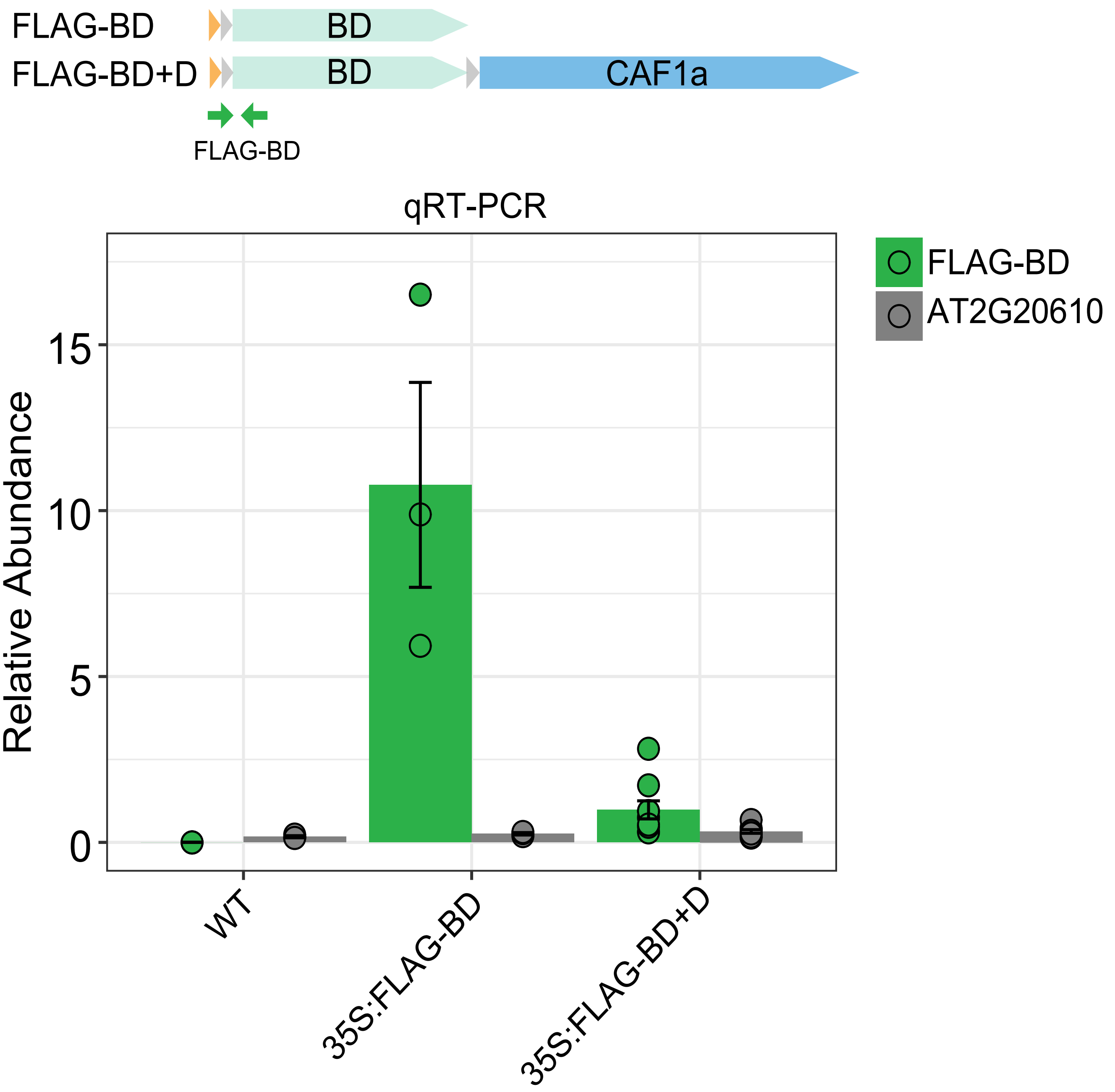

Supplemental Figure 4

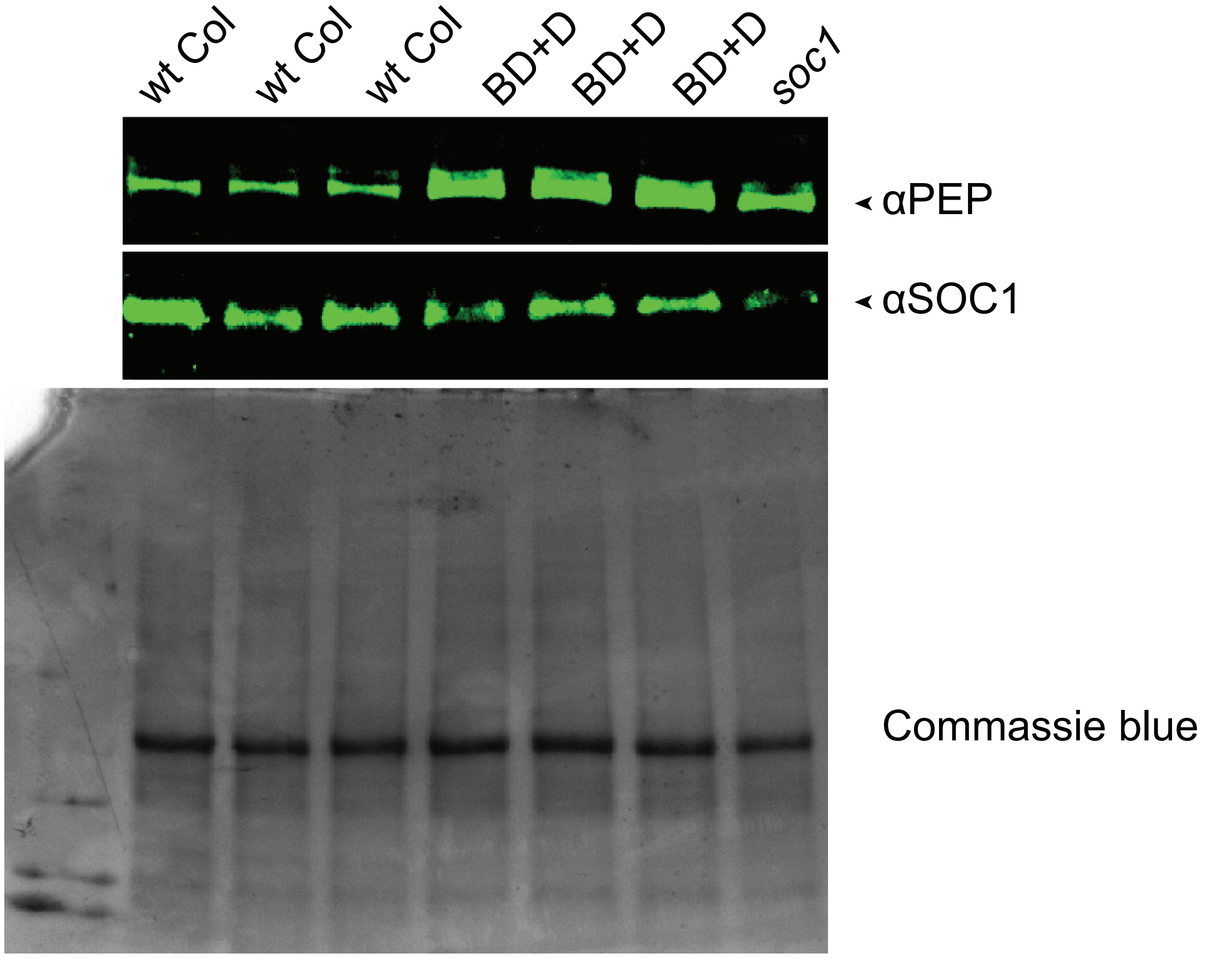

Supplemental Figure 5

(A) FLAG-BD

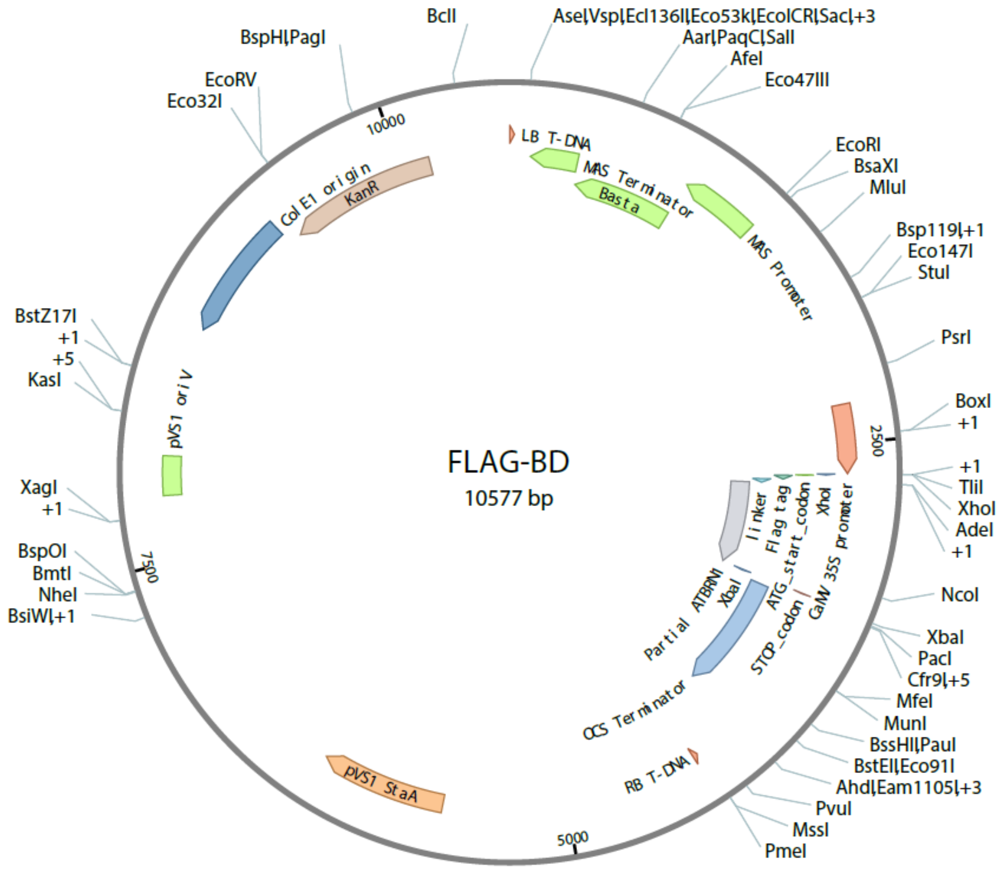

> Flag-BD DNA sequence

atggattataaggacgatgacgataaaggcatccacggggtgccagctgctGCGGAAGCGAAGGAGGAGAATAGGGAGAAAAACGAAG
AAGAAGAGAGCGTAAAGCTTTTCGTCGGACAAATTCCGAAACATATGTCGGAATCTCAACTCTTAACATTGTTTCA
AGAGTTCGCTGTCGTTGACGAGGTCAATATCATCAAGGACAAGATTACACGCGCCTCACGAGGATGTTGTTTTCT
GCTATGTCCATCGAGAGAAGAAGCAGATAAGTTGGTCAATGCTTGCCATAACAAGAAGACATTGCCTGGGGCTA
ACAGTCTATTGCAAGTAAAGTATGCAGATGGCGAATTAGAAAGGCTAGAGCATAAGCTATTTGTTGGTATGCTTC
CAAAGAATGTCTCTGAAGCTGAAGTTCAATCTTTATTCTCCAAGTACGGGACCATAAAGGATCTACAGATTCTAAG
AGGTGCTCAACAAACGAGCAAAGGCTGTGCATTTCTGAAGTATGAGACAAAAGAACAAGCTGTTTCCGCCATGG
AATCTATCAACGGAAAACATAAAATGGAGGGTTCAACTGTTCCTTTAGTTGTCAAATGGGCAGACACAGAACGAG
AAAGACACACAAGAAGACTTCAAAAGGCTCAATAA

> FLAG-BD amino acid sequence

MDYKDDDDKGIHGVPAAAEAKEENREKNEEEESVKLFVGQIPKHMSESQLLTLFQEFAVVDEVNIIKDKITRASRGCC
FLLCPSREEADKLVNACHNKKTLPGANSLLQVKYADGELERLEHKLFVGMLPKNVSEAEVQSLFSKYGTIKDLQILRG
AQQTSKGCAFLKYETKEQAVSAMESINGKHKMEGSTVPLVVKWADTERERHTRRLQKAQ*

ATG FLAG Linker BD STOP

(B) BD+D

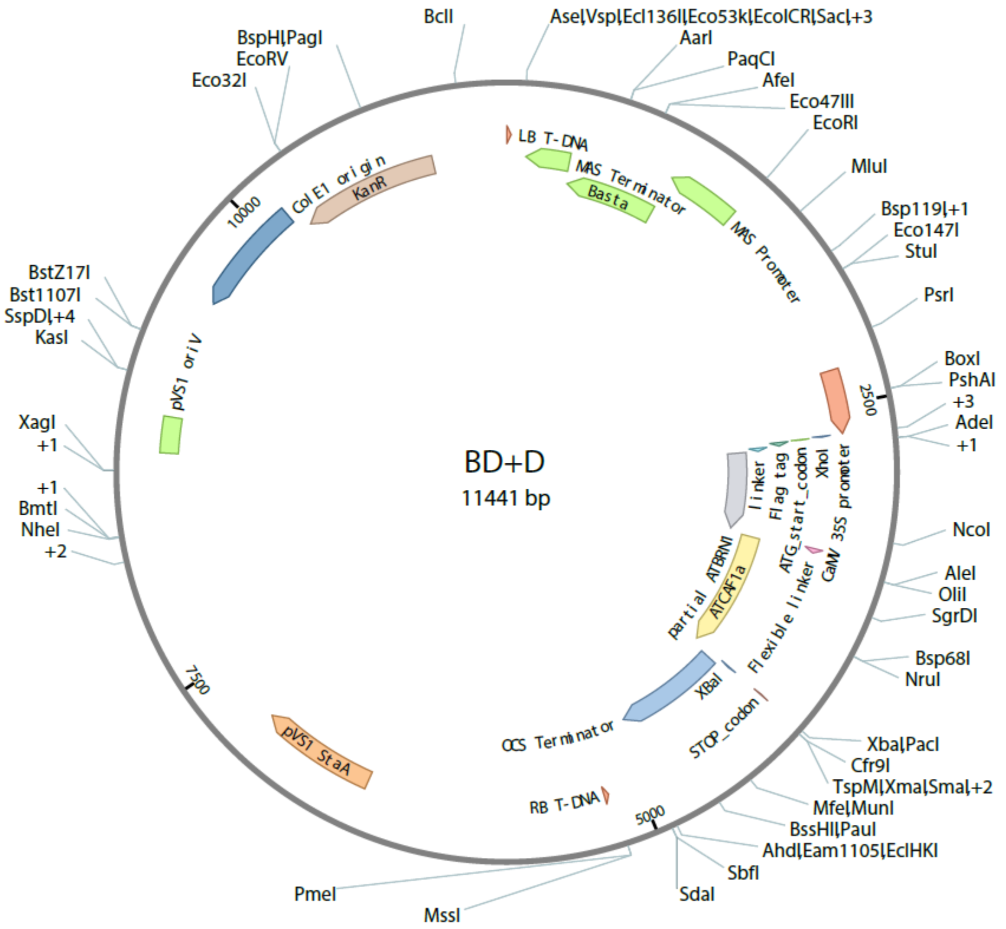

> Flag BD+D DNA sequence

atggattataaggacgatgacgataaaggcatccacggggtgccagctgctGCGGAAGCGAAGGAGGAGAATAGGGAGAAAAACGAAG
AAGAAGAGAGCGTAAAGCTTTTCGTCGGACAAATTCCGAAACATATGTCGGAATCTCAACTCTTAACATTGTTTCA
AGAGTTCGCTGTCGTTGACGAGGTCAATATCATCAAGGACAAGATTACACGCGCCTCACGAGGATGTTGTTTTCT
GCTATGTCCATCGAGAGAAGAAGCAGATAAGTTGGTCAATGCTTGCCATAACAAGAAGACATTGCCTGGGGCTA
ACAGTCTATTGCAAGTAAAGTATGCAGATGGCGAATTAGAAAGGCTAGAGCATAAGCTATTTGTTGGTATGCTTC
CAAAGAATGTCTCTGAAGCTGAAGTTCAATCTTTATTCTCCAAGTACGGGACCATAAAGGATCTACAGATTCTAAG
AGGTGCTCAACAAACGAGCAAAGGCTGTGCATTTCTGAAGTATGAGACAAAAGAACAAGCTGTTTCCGCCATGG
AATCTATCAACGGAAAACATAAAATGGAGGGTTCAACTGTTCCTTTAGTTGTCAAATGGGCAGACACAGAACGAG
AAAGACACACAAGAAGACTTCAAAAGGCTCAAggaggatctggaggaggatctggaggaGCGATCATTAAACCAAACCGAGA
TCTCAAACCGGACGGTGTCACAGTCGTGACTCGTGAAGTCTGGGCGGAGAATCTCGAATCAGAATTTGAACTAA
TCAGCGAAATCATCGACGATTATCCATTTATCTCAATGGACACAGAATTTCCCGGCGTGATTTTCAAATCCGATCT
CCGGTTTACGAATCCCGACGATCTTTATACTCTTCTTAAAGCTAACGTCGACGCTCTCAGCTTAATCCAAGTCGG
TCTCACTCTCTCTGATGTTAACGGTAACCTCCCAGACCTCGGTGACGATCTTCACCGAGGATTCATCTGGGAGTT
TAATTTCCGTGACTTCGACGTGGCGCGTGACGCACACGCGCCTGATTCGATCGAGTTGCTTCGTCGGCAAGGTA
TCGATTTTGAGCGGAATTGTCGCGACGGTGTTGAGTCGGAGAGGTTTGCGGAGCTGATGATGTCGTCGGGGCT
TGTTTGTAATGAGGAAGTGAGCTGGGTGACGTTTCATAGCGCGTATGATTTCGGTTACTTGATGAAGATTCTCAC
GCGCCGAGAATTGCCCGGCGCGTTGGGGGAGTTTAAGCGCGTGATGAGAGTGTTGTTTGGGGAGCGTGTGTAT
GATGTGAAGCATATGATGAAGTTTTGTGAAAGGAGATTGTTTGGTGGTTTGGACCGGGTTGCAAGGACGCTTGA

GGTTAACCGGGCGGTTGGGAAATGTCATCAAGCTGGTTCGGATAGTTTGCTTACGTGGCATGCGTTTCAGAGGA
TGAGGGATTTGTATTTTGTTCAAGATGGGCCGGAGAAACATGCTGGGGTTTTGTATGGGCTTGAGGTTTTTTAA

>Flag BD+Deadenylase amino acid sequence

MDYKDDDDKGIHGVPAAAEAKEENREKNEEEESVKLFVGQIPKHMSESQLLTLFQEFAVVDEVNIIKDKITRASRGCC
FLLCPSREEADKLVNACHNKKTLPGANSLLQVKYADGELERLEHKLFVGMLPKNVSEAEVQSLFSKYGTIKDLQILRG
AQQTSKGCAFLKYETKEQAVSAMESINGKHKMEGSTVPLVVKWADTERERHTRRLQKAQGGSGGGSGGAIIKPNRD
LKPDGVTVVTREVWAENLESEFELISEIIDDYPFISMDTEFPGVIFKSDLRFTNPDDLYTLLKANVDALSLIQVGLTLSDV
NGNLPDLGDDLHRGFIWEFNFRDFDVARDAHAPDSIELLRRQGIDFERNCRDGVESERFAELMMSSGLVCNEEVSW
VTFHSAYDFGYLMKILTRRELPGALGEFKRVMRVLFGERVYDVKHMMKFCERRLFGGLDRVARTLEVNRAVGKCHQ
AGSDSLLTWHAFQRMRDLYFVQDGPEKHAGVLYGLEVF*

ATG FLAG Linker 1 BD Flexible Linker 2 AtCAF1a - (AT3G44260) mRNA deadenylase protein STOP

(C) BD +R

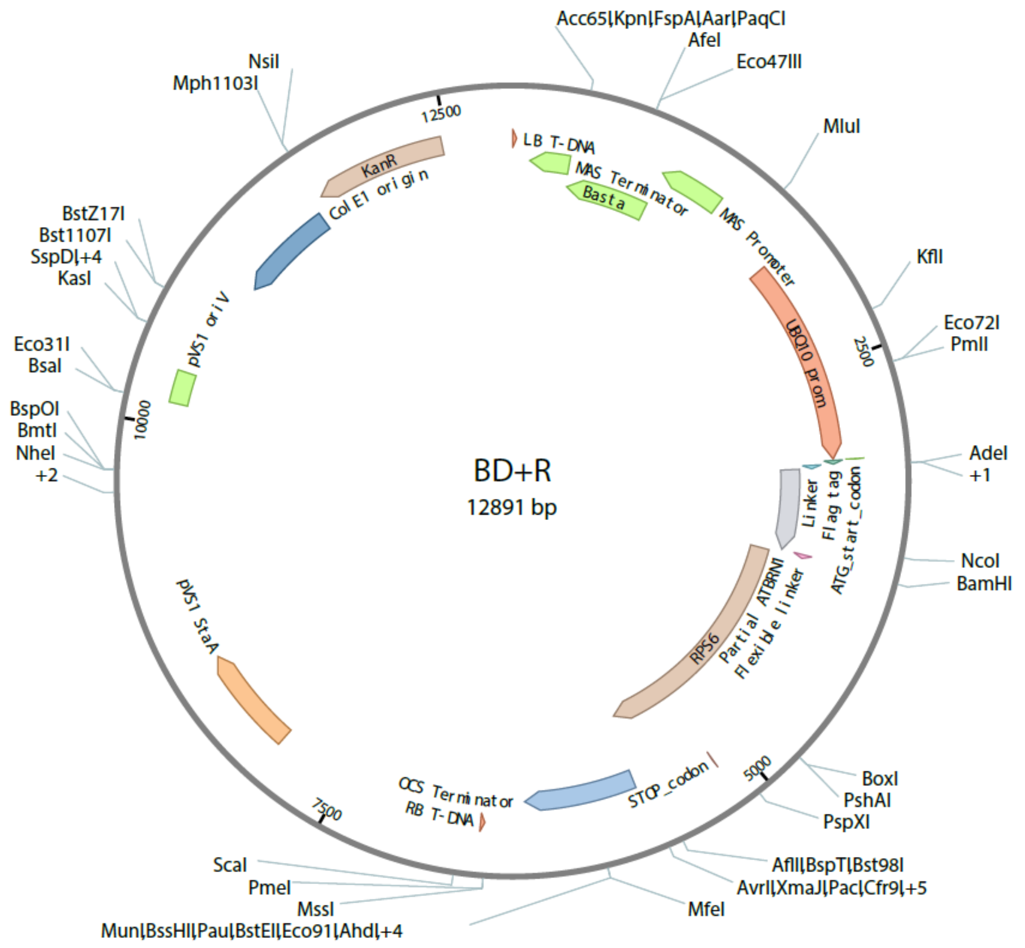

>Flag BD-RPS6 DNA Sequence

GATCAGGATATTCTTGTTTAAGATGttgaactctatggaggtttgtatgaactgatgatctaggaccggataagttcccttcttcatagcgaacttattcaa
agaatgttttgtgtatcattcttgttacattgttattaatgaaaaaatattattggtcattggactgaacacgagtgttaaatatggaccaggccccaaataagatccattgata
tatgaattaaataacaagaataaatcgagtcaccaaaccacttgccttttttaacgagacttgttcaccaacttgatacaaaagtcattatcctatgcaaatcaataatcat
acaaaaatatccaataacactaaaaaattaaaagaaatggataatttcacaatatgttatacgataaagaagttacttttccaagaaattcactgattttataagcccact
tgcattagataaatggcaaaaaaaaacaaaaaggaaaagaaataaagcacgaagaattctagaaaatacgaaatacgcttcaatgcagtgggacccacggttc
aattattgccaattttcagctccaccgtatatttaaaaaataaaacgataatgctaaaaaaatataaatcgtaacgatcgttaaatctcaacggctggatcttatgacgac
cgttagaaattgtggttgtcgacgagtcagtaataaacggcgtcaaagtggttgcagccggcacacacgagtcgtgtttatcaactcaaagcacaaatacttttcctca
acctaaaaataaggcaattagccaaaaacaactttgcgtgtaaacaacgctcaatacacgtgtcattttattattagctattgcttcaccgccttagctttctcgtgacctag
tcgtcctcgtcttttcttcttcttcttctataaaacaatacccaaagagctcttcttcttcacaattcagatttcaatttctcaaaatcttaaaaactttctctcaattctctctaccgtg
atcaaggtaaatttctgtgttccttattctctcaaaatcttcgattttgttttcgttcgatcccaatttcgtatatgttctttggtttagattctgttaatcttagatcgaagacgattttctg
ggtttgatcgttagatatcatcttaattctcgattagggtttcatagatatcatccgatttgttcaaataatttgagttttgtcgaataattactcttcgatttgtgatttctatctagatc
tggtgttagtttctagtttgtgcgatcgaatttgtcgattAATCTGAGTTTTTCTGATTAACAGGCTCGAGATGGATTATAaggacgatgacgat
aaaggcatccacggggtgccagctgctGCGGAAGCGAAGGAGGAGAATAGGGAGAAAAACGAAGAAGAAGAGAGCGTAAA
GCTTTTCGTCGGACAAATTCCGAAACATATGTCGGAATCTCAACTCTTAACATTGTTTCAAGAGTTCGCTGTCGTT
GACGAGGTCAATATCATCAAGGACAAGATTACACGCGCCTCACGAGGATGTTGTTTTCTGCTATGTCCATCGAGA
GAAGAAGCAGATAAGTTGGTCAATGCTTGCCATAACAAGAAGACATTGCCTGGGGCTAACAGTCTATTGCAAGTA
AAGTATGCAGATGGCGAATTAGAAAGGCTAGAGCATAAGCTATTTGTTGGTATGCTTCCAAAGAATGTCTCTGAA
GCTGAAGTTCAATCTTTATTCTCCAAGTACGGGACCATAAAGGATCTACAGATTCTAAGAGGTGCTCAACAAACG

AGCAAAGGCTGTGCATTTCTGAAGTATGAGACAAAAGAACAAGCTGTTTCCGCCATGGAATCTATCAACGGAAAA
CATAAAATGGAGGGTTCAACTGTTCCTTTAGTTGTCAAATGGGCAGACACAGAACGAGAAAGACACACAAGAAGA
CTTCAAAAGGCTCAAggaggatctggaggaggATCTGGAGGAGGATCCAAGGTGCGTGACGGCGAAATCgaatagactctac
atttgctcgtattcgagtttcctttgctaaccttttcatgtttcttcgtttcagTTCAACGTTGCGAATCCAACTACTGGATGCCAGAAGAAGCTC
GAGATCGACGATGACCAGAAACTgtaagattttgcttctctgtttcatcttcattcatttttaatagatatttcgtgctaggttgccagatctattgattggtgtttgt
atatatcactgttaaattgataggtctagtaaacagtaattgtcaatttttctatgttcgtcttataagcttaataagagttagaatcttcttctatgtttaatacttacattaaaata
agtttcgtatctgctcataaacatatctggattatattattcagcttgatttatgttgatgcagACGTGCGTTTTACGACAAGAGAATCTCTCAAGAAG
TCAGTGGAGATGCTTTGGGCGAGgtacagattccatttttcttactttgatctaatgagtgtgaagattagcatttcgaagcagatgatgatgttttgtttatga
attgttgttattgcagGAGTTCAAAGGATACGTTTTCAAGATCAAGGGTGGTTGCGATAAGCAAGGTTTCCCAATGAAGCA
GGGAGTTTTGACTCCAGGCCGTGTTCGCCTTTTGCTTCACCGAGgtaactaaacataatacctttttcttacacttttgcatctataatggatt
tgtatttgtgtgtgtgtgtgttttgctgtagattaacattgtttcgtaatgggtgaacagGAACTCCTTGCTTCAGAGGACATGGAAGGAGAACTGG
TGAGAGGAGAAGAAAGTCTGTTCGTGGTTGCATTGTGAGCCCTGATCTCTCTGTTCTGAACCTTGTCATTGTGAA
GAAGGGTGAGAACGATCTTCCTGGGCTTACCGATACTGAGAAGCCAAGAATGAGAGGACCAAAGAGAGCCTCC
AAGATCCGTAAACTGTTTAACCTCAAGAAGGAAGATGATGTCAGGACCTATGTCAACACTTACCGCCGCAAGTTC
ACAAACAAGAAGGgtgagacactgaacttgagaaatagtggtttctgaatcacacttgagaaataatgttttctgaaacttgtttcttctgtttggatttttgacagG
CAAGGAAGTTAGCAAAGCCCCTAAGATCCAGAGGCTTGTGACCCCATTGACTCTTCAGAGGAAGAGAGCTAGAA
TTGCTGACAAGAAGAAGAAAATTGCTAAGGCTAATTCTGATGCTGCTGATTACCAGAAGCTTCTCGCCTCGAGGT
TGAAGGAACAGCGTGACAGGAGGAGTGAGAGTTTGGCAAAGAAGAGGTCGAGACTCTCTTCTGCTGCTGCCAA
GCCCTCTGTCACAGCTTAAaaaagcttgagaatcaaatcaaagatgtcacctttctagttttgttctcttttcttctccggctattaaaaagttagttcgagttcat
attcagctgtttttgttccaagacatgggtacacaatcgctttgggcttgtttcatgatttatgcttttagttgaagaccattttaaactcaatttttattgcttatctttctcagtttttgat
caaagcgtttctccattgaaaaccattttaatcacctgtttcaatcatacataatgttaaacacatgtactggcgattacaataggtatatattttgactgaaatttatggagca
ttgtatgacggtttgataaatttctgagaaaactcattcttaagttttaagattatctcaacaactggtgaatcaaaactgtgatgtgtggtttGGGCCAAACGACCC
ATAAACGCCTAGGTCTAGAGAGTTAATTAAGACCCGGGACTAGTCCCTAGAGTCCTGCTTTAATGAGATATGCGA
GACGCCTATGATCGCATGATATTTGCTTTCAATTCTGTTGTGCACGTTGTAAAAAACCTGAGCATGTGTAGCTCAG
ATCCTTACCGCCGGTTTCGGTTCATTCTAATGAATATATCACCCGTTACTATCGTATTTTTATGAATAATATTCTCC
GTTCAATTTACTGATTGTACCCTACTACTTATATGTACAATATTAAAATGAAAACAATATATTGTGCTGAATAGGTT
TATAGCGACATCTATGATAGAGCGCCACAATAACAAACAATTGCGTTTTATTATTACAAATCCAATTTTAAAAAAAG
CGGCAGAACCGGTCAAACCTAAAAGACTGATTACATAAATCTTATTCAAATTTCAAAAGTGCCCCAGGGGCTAGT
ATCTACGACACACCGAGCGGCGAACTAATAACGCTCACTGAAGGGAACTCCGGTTCCCCGCCGGCGCGCATGG
GTGAGATTCCTTGAAGTTGAGTATTGGCCGTCCGCTCTACCGAAAGTTACGGGCACCATTCAACCCGGTCCAGC
ACGGCGGCCGGGTAACCGACTTGCTGCCCCGAGAATTATGCAGCATTTTTTTGGTGTATGTGGGCCCCAAATGA
AGTGCAGGTCAAACCTTGACAGTGACGACAAATCGTTGGGCGGGTCCAGGGCGAATTTTGCGACAACATGTCGA
GGCTCAGCAG

UBQ10 promoterATGFLAGLinker 1 BDFlexible Linker 2 Genomic *RPS6*
(AT4G31700)STOPOCS terminator

(D) 5’BD - Flag BD MCS vector (UBQ10-ATG-Flag BD-MCS-OCS)

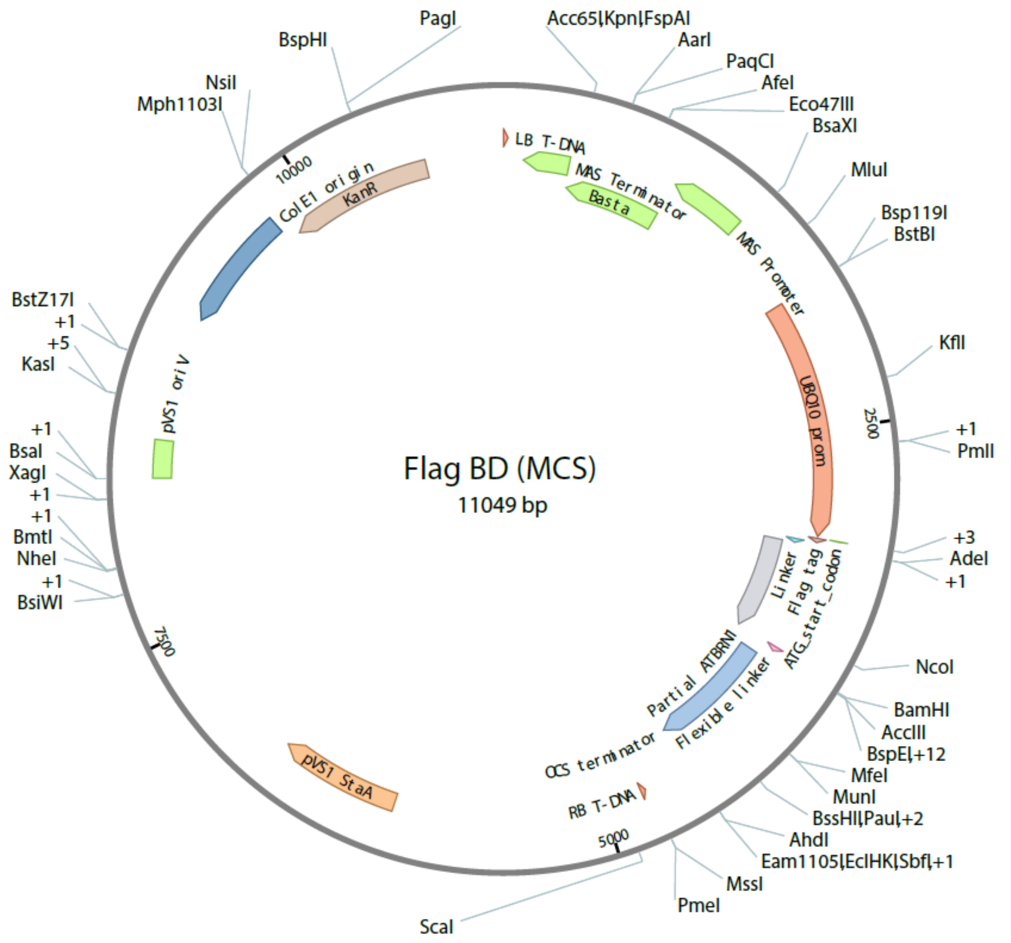

>UBQ10-ATG-Flag BD-MCS-OCS DNA Sequence

GATCAGGATATTCTTGTTTAAGATGttgaactctatggaggtttgtatgaactgatgatctaggaccggataagttcccttcttcatagcgaacttattcaa
agaatgttttgtgtatcattcttgttacattgttattaatgaaaaaatattattggtcattggactgaacacgagtgttaaatatggaccaggccccaaataagatccattgata
tatgaattaaataacaagaataaatcgagtcaccaaaccacttgcctttttaacgagacttgttcaccaacttgatacaaaagtcattatcctatgcaaatcaataatcat
acaaaaatatccaataacactaaaaaattaaaagaaatggataatttcacaatatgttatacgataaagaagttacttttccaagaaattcactgattttataagcccact
tgcattagataaatggcaaaaaaaaacaaaaaggaaaagaaataaagcacgaagaattctagaaaatacgaaatacgcttcaatgcagtgggacccacggttc
aattattgccaattttcagctccaccgtatatttaaaaaataaaacgataatgctaaaaaaatataaatcgtaacgatcgttaaatctcaacggctggatcttatgacgac
cgttagaaattgtggttgtcgacgagtcagtaataaacggcgtcaaagtggttgcagccggcacacacgagtcgtgtttatcaactcaaagcacaaatacttttcctca
acctaaaaataaggcaattagccaaaaacaactttgcgtgtaaacaacgctcaatacacgtgtcattttattattagctattgcttcaccgccttagctttctcgtgacctag
tcgtcctcgtcttttcttcttcttcttctataaaacaatacccaaagagctcttcttcttcacaattcagatttcaatttctcaaaatcttaaaaactttctctcaattctctctaccgtg
atcaaggtaaatttctgtgttccttattctctcaaaatcttcgattttgttttcgttcgatcccaatttcgtatatgttctttggtttagattctgttaatcttagatcgaagacgattttctg
ggtttgatcgttagatatcatcttaattctcgattagggtttcatagatatcatccgatttgttcaaataatttgagttttgtcgaataattactcttcgatttgtgatttctatctagatc
tggtgttagtttctagtttgtgcgatcgaatttgtcgattAATCTGAGTTTTTCTGATTAACAGGCTCGAGATGGATTATAaggacgatgacgat
aaaggcatccacggggtgccagctgctGCGGAAGCGAAGGAGGAGAATAGGGAGAAAAACGAAGAAGAAGAGAGCGTAAA
GCTTTTCGTCGGACAAATTCCGAAACATATGTCGGAATCTCAACTCTTAACATTGTTTCAAGAGTTCGCTGTCGTT
GACGAGGTCAATATCATCAAGGACAAGATTACACGCGCCTCACGAGGATGTTGTTTTCTGCTATGTCCATCGAGA
GAAGAAGCAGATAAGTTGGTCAATGCTTGCCATAACAAGAAGACATTGCCTGGGGCTAACAGTCTATTGCAAGTA
AAGTATGCAGATGGCGAATTAGAAAGGCTAGAGCATAAGCTATTTGTTGGTATGCTTCCAAAGAATGTCTCTGAA
GCTGAAGTTCAATCTTTATTCTCCAAGTACGGGACCATAAAGGATCTACAGATTCTAAGAGGTGCTCAACAAACG
AGCAAAGGCTGTGCATTTCTGAAGTATGAGACAAAAGAACAAGCTGTTTCCGCCATGGAATCTATCAACGGAAAA

CATAAAATGGAGGGTTCAACTGTTCCTTTAGTTGTCAAATGGGCAGACACAGAACGAGAAAGACACACAAGAAGA
CTTCAAAAGGCTCAAggaggatctggaggaggatctggaggaggatcctccggacctaggtctagaGAGTTAATTAAGACCCGGGACTA
GTCCCTAGAGTCCTGCTTTAATGAGATATGCGAGACGCCTATGATCGCATGATATTTGCTTTCAATTCTGTTGTGC
ACGTTGTAAAAAACCTGAGCATGTGTAGCTCAGATCCTTACCGCCGGTTTCGGTTCATTCTAATGAATATATCACC
CGTTACTATCGTATTTTTATGAATAATATTCTCCGTTCAATTTACTGATTGTACCCTACTACTTATATGTACAATATT
AAAATGAAAACAATATATTGTGCTGAATAGGTTTATAGCGACATCTATGATAGAGCGCCACAATAACAAACAATTG
CGTTTTATTATTACAAATCCAATTTTAAAAAAAGCGGCAGAACCGGTCAAACCTAAAAGACTGATTACATAAATCTT
ATTCAAATTTCAAAAGTGCCCCAGGGGCTAGTATCTACGACACACCGAGCGGCGAACTAATAACGCTCACTGAA
GGGAACTCCGGTTCCCCGCCGGCGCGCATGGGTGAGATTCCTTGAAGTTGAGTATTGGCCGTCCGCTCTACCG
AAAGTTACGGGCACCATTCAACCCGGTCCAGCACGGCGGCCGGGTAACCGACTTGCTGCCCCGAGAATTATGC
AGCATTTTTTTGGTGTATGTGGGCCCCAAATGAAGTGCAGGTCAAACCTTGACAGTGACGACAAATCGTTGGGC
GGGTCCAGGGCGAATTTTGCGACAACATGTCGAGGCTCAGCAG

UBQ10 promoterATGFLAGLinker 1 BDFlexible Linker 2
MCS(BamHI,MroI,AvrII,XmaI,SpeI)OCS terminator

**Insert needs STOP codon

Supplemental Figure 6

From Figure 1B

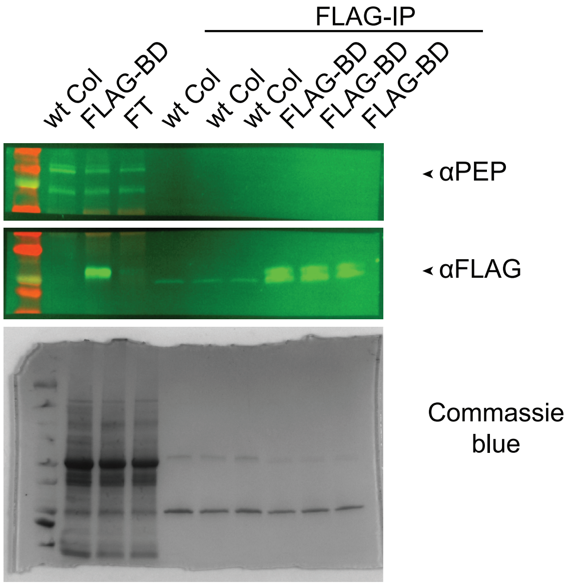

From Figure 2C

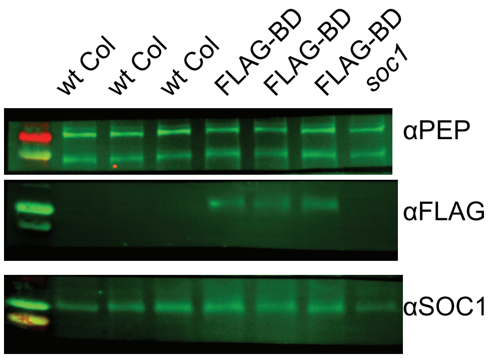

From Figure 5D

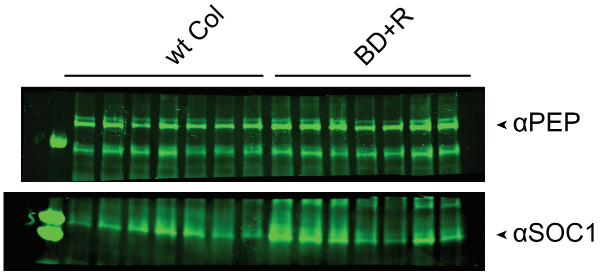

From Figure S4

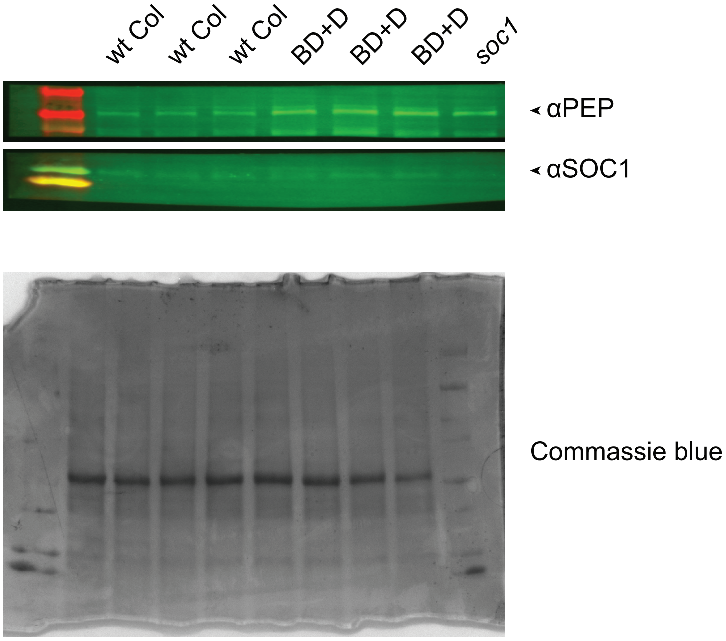

Supplement: Supplementary file 1 — Additional file 1: Figure S1. FLAG-BD interacts with other mRNAs in addition to SOC1. Extended analysis of the experiment from Figure 1C for three additional mRNAs that have the identical 7-nt BRN1 binding site sequence. Each biological replicate is shown as a point. The bar represents the average and error bars represent the standard deviation between three biological replicates. Figure S2. IP control gels performed before Mass Spectrometry. (A) Coomassie blue stained SDS-PAGE gels of the mock-IP (left) and four biological replicates (R1-R4) of the FLAG-IP (right). IN = input protein sample, FT = flow through sample that did not interact with the beads or FLAG antibody. (B) Western blot of the samples in part 'A'. The PEPC protein is not detected in the mock-IP or FLAG-IPs (top). The FLAG-BD protein is detected in the FLAG-IPs but not mock-IP (bottom). Arrowheads denote the expected size of the proteins detected. Figure S3. Reduced expression of the BD+D transgene. qRT-PCR of the relative mRNA accumulation of the FLAG-BD and BD+D transgenes. AT2G20610 is a constitutively-expressed control gene. At least three biological replicates for each genotype were used (shown as points), the height of the bar represents their average and the error bars represent the standard deviation. The transgene structure and position of the RT-PCR primers is shown above. Figure S4. SOC1 protein accumulation in BD+D plants. Western blot displaying SOC1 protein levels with the BD+D transgene. The three wt Col and BD+D samples are biological replicates. PEP is an unrelated protein used as a loading control. Arrowheads mark the predicted size of the protein detected. Quantification of this Western blot is shown in Figure 4F. Figure S5. Plasmid maps and sequences. Vector maps and annotated plasmid sequences of (A) FLAG-BD, (B) BD+D, (C) BD+R and (D) a vector with multiple cloning site (MCS) to fuse any protein to AtUBQ10:FLAG-BD. Figure S6. Full Western blots from other figures. The full un-c [file 13007_2022_907_MOESM1_ESM.pdf]
