# Supplementary material for: A plant tethering system for the functional study of protein-RNA interactions in vivo
Source: Plant Methods. 2022 Jun 4;18:75. doi: 10.1186/s13007-022-00907-w (PMC9166424; doi:10.1186/s13007-022-00907-w)
Supplement: Supplementary file 2 — Additional file 2: Table S1. Mass Spectrometry data of the 20 proteins identified in at least two biological replicates. [file 13007_2022_907_MOESM2_ESM.pdf]

Supplemental Table 1

Mass Spectrometry data of the 20 proteins identified in at least two biological replicates

			Weighted spectral Count					Replicate 1/ Mock		Replicate 2/ Mock		Replicate 3/ Mock		Replicate 4/ Mock	
**Locus**	**Description**	**GO category in Figure 3B (1: RNA binding, 2: Linked to RNA biology, 3: Not linked to RNA)**	**Mock**	**Replicate 1**	**Replicate 2**	**Replicate 3**	**Replicate 4**	**Log2(FC)**	**NegLog(p)**	**Log2(FC)**	**NegLog(p)**	**Log2(FC)**	**NegLog(p)**	**Log2(FC)**	**NegLog(p)**
AT1G54410	Dehydrin family protein	1	0	6	7	6	9	INF	1.86097	INF	1.658705	INF	0.87786	INF	1.448106
AT1G67700	Multidrug resistance protein	1	0	8	6	12	14	INF	2.481909	INF	1.421615	INF	1.756316	INF	2.253312
AT1G79040	Photosystem II subunit R (PSBR)	1	0	4	7	10	9	INF	1.240339	INF	1.658705	INF	1.463431	INF	1.448106
AT2G19750	Ribosomal protein S30 family protein	1	0	5	12	8	8	INF	1.550616	INF	2.844818	INF	1.170612	INF	1.287125
AT2G33530	Serine carboxypeptidase-like 46 (SCPL46)	1	0	5	6	5	8	INF	1.550616	INF	1.421615	INF	0.731508	INF	1.287125
AT2G38140	Plastid-specific ribosomal protein 4 (PSRP4)	1	0	9	10	9	13	INF	2.792494	INF	2.37024	INF	1.317013	INF	2.092231
AT2G41740	Villin 2(VLN2)	1	0	3	6	13	11	INF	0.930139	INF	1.184569	INF	1.756316	INF	1.609107
AT3G57410	Villin 3(VLN3)	1	0	2	9	13	11	INF	0.620016	INF	2.133018	INF	1.902783	INF	1.770128
AT4G30160	Villin 4(VLN4)	1	0	4	8	16	14	INF	0.930139	INF	1.658705	INF	2.195768	INF	2.092231
AT5G38430	Ribulose bisphosphate carboxylase (small chain) family protein (RBCS1B)	1	0	0	0	71	77	NaN	0	NaN	0	INF	1.317013	INF	1.448106
AT5G47210	Hyaluronan / mRNA binding family	1	0	6	10	13	12	INF	1.86097	INF	2.37024	INF	1.902783	INF	1.93117
AT5G26000	thioglucoside glucohydrolase 1 (TGG1)	2	29	25	47	112	104	-0.214125	0.400125	0.696608	0.544525	1.949374	1.725202	1.842459	1.920893
AT3G25230	Rotamase FKBP 1 (ROF1)	3	0	7	10	10	9	INF	2.171401	INF	2.37024	INF	1.463431	INF	1.448106
AT4G35450	Ankyrin repeat-containing protein 2 (AKR2)	3	0	5	4	10	9	INF	1.550616	INF	0.947567	INF	1.463431	INF	1.448106
AT4G35630	Phosphoserine aminotransferase (PSAT)	3	2	17	18	7	7	3.087463	3.583188	3.169925	2.679162	1.807355	0.300372	1.807355	0.358798
AT5G64040	Photosystem I reaction center subunit PSI-N	3	0	10	12	9	9	INF	3.103157	INF	2.844818	INF	1.317013	INF	1.448106
AT2G03440	Nodulin-related protein 1 (NRP1)	3	3	12	17	10	11	2	1.843168	2.5025	1.999587	1.736966	0.332408	1.874469	0.485876
AT1G33600	Leucine-rich repeat (LRR) family protein	3	0	5	14	17	14	INF	1.550616	INF	3.319573	INF	2.488819	INF	2.253312
AT3G12145	Leucine-rich repeat (LRR) family protein	3	0	2	8	11	6	INF	0.620016	INF	1.895839	INF	1.609865	INF	0.965224
AT4G03520	Thioredoxin superfamily protein (ATHM2)	3	2	3	9	21	17	0.584963	0.317946	2.169925	1.027264	3.392318	1.660553	3.087463	1.41504
