# Supplementary material for: A plant tethering system for the functional study of protein-RNA interactions in vivo
Source: Plant Methods. 2022 Jun 4;18:75. doi: 10.1186/s13007-022-00907-w (PMC9166424; doi:10.1186/s13007-022-00907-w)
Supplement: Supplementary file 3 — Additional file 3: Table S2. Primer sequences and alleles used in this study. [file 13007_2022_907_MOESM3_ESM.pdf]

Supplemental Table 2. Primer sequences and alleles used in this report

Primers and sequences used in this report

Figure	Experiment	Target	Forward primer sequence	Reverse primer sequence
1C, S3	RIP qRT-PCR, qRT-PCR	Control gene AT2G20610	TyrAT qRT F3 - AAAGTTGGAGCTGTCATTGATGG	TyrAT qRT R3- AACACGAGATTCTCCTCTCTGGC
1C, 2B, 4C, 5F, S1	RIP qRT-PCR, qRT-PCR	SOC1	soc1 qRT F - TGGGGATCTCATGAAAGCGAA	soc1 qRT R - ACCCAATGAACAATTGCGTCTC
1C, 2B, 4C, 5F, S1, S3	RIP qRT-PCR, qRT-PCR	Control gene AT1G08200	AT1G08200 qRT F3- agacgtcgctttgggacttg	AT1G08200 qRT R3- atgcgactggttttgaagtgg
4D, 5G	qRT-PCR	SOC1 intron-exon	soc1 intron-exon qRT F - GTTTTGAGAACACCATTGGGAT	soc1 intron-exon qRT R - TTTGGTGCTGACTCGATCCTT
4D, 5G	qRT-PCR	AT1G08200 intron-exon	AT1G02800 intron-exon qRT F- CGAATTTCGAATTGTGGATGTCT	AT1G02800 intron-exon qRT R- TGCTGGAGTACAGATCGCAG
4E	ePAT	ePAT Control	GCGAGCTCCGCGGCCGCGTTTTTTTTTTTTVN	none
4E	ePAT	ePAT anchor primer	GCGAGCTCCGCGGCCGCGTTTTTTTTTTTT	none
4E	ePAT	soc1 qRT 3UTR II F	CAAGTAACAAAACTCTAAAGCTGC	none
4E	ePAT	soc1 qRT 3UTR III F	TCTATGTTCTTACACTTGCCTCTCT	none
5A	pEarlyGate100 promoter swap 35S->UBQ10	UBQ10 promoter	UBQ10 Prom F inf_2 - TAGGAAGGAAGTTCGAAGATCAGGATATTCTTGTTTAAGATG	UBQ10 Prom R inf_2 - TATAATCCATCTCGAGCCTGTTAATCAGAAAAACTCAGATT
5A	BD-RPS6 construct	genomic RPS6	RPS6 noATG Stop F2 - ATCTGGAGGAGGATCCAAGGTGCGTGACGGCGAAATC	RPS6 noATG Stop R2 - ACTCTCTAGACCTAGGCGTTTATGGGTCGTTTGGCCC
S1	RIP qRT-PCR	AT2G22125	AT2G22125_qRT_F - TCATATTTGTTCATCTTTTGGTGTGCC	AT2G22125_qRT_R - ATGCTGACCAAGAGGACTTAGGC
S1	RIP qRT-PCR	AT1G69640	AT1G69640_qRT_F - GGAACTTACATGCCTTACTCGC	AT1G69640_qRT_R - TCCATTTCTCCAACCTCAGGG
S1	RIP qRT-PCR	AT2G33370	AT2G33370_qRT_F - GATTGCTAGTGCTGCTAACGC	AT2G33370_qRT_R - ACACCAACTATTTCGCGTTTCG
S3	qRT-PCR	FLAG-BD and BD+D	q_FLAG_BD_F - TAAAGGCATCCACGGGGTGCCA	q_FLAG_BD_R - TTGTCCGACGAAAAGCTTTACGC

Mutant alleles used in this report

Figure	Mutant	Allele	Background Ecotype
1C, 2A-D, 4B-D, 4F, 5F-G, S1, S4	*soc1*	*soc1-6* (SALK_138131)	Col
2A	*brn1*	*atbrn1* (SALK_041205)	Col
